# Co-Expression of α9β1 Integrin and VEGF-D Confers Lymphatic Metastatic Ability to a Human Breast Cancer Cell Line MDA-MB-468LN

**DOI:** 10.1371/journal.pone.0035094

**Published:** 2012-04-24

**Authors:** Mousumi Majumder, Elena Tutunea-Fatan, Xiping Xin, Mauricio Rodriguez-Torres, Jose Torres-Garcia, Ryan Wiebe, Alexander V. Timoshenko, Rabindra N. Bhattacharjee, Ann F. Chambers, Peeyush K. Lala

**Affiliations:** 1 Department of Anatomy and Cell Biology, The University of Western Ontario, London, Ontario, Canada; 2 Department of Biology, The University of Western Ontario, London, Ontario, Canada; 3 Department of Medical Biophysics, The University of Western Ontario, London, Ontario, Canada; 4 Department of Oncology, The University of Western Ontario, London, Ontario, Canada; Ottawa Hospital Research Institute, Canada

## Abstract

**Introduction and Objectives:**

Lymphatic metastasis is a common occurrence in human breast cancer, mechanisms remaining poorly understood. MDA-MB-468LN (468LN), a variant of the MDA-MB-468GFP (468GFP) human breast cancer cell line, produces extensive lymphatic metastasis in nude mice. 468LN cells differentially express α9β1 integrin, a receptor for lymphangiogenic factors VEGF-C/-D. We explored whether (1) differential production of VEGF-C/-D by 468LN cells provides an autocrine stimulus for cellular motility by interacting with α9β1 and a paracrine stimulus for lymphangiogenesis *in vitro* as measured with capillary-like tube formation by human lymphatic endothelial cells (HMVEC-dLy); (2) differential expression of α9 also promotes cellular motility/invasiveness by interacting with macrophage derived factors; (3) stable knock-down of VEGF-D or α9 in 468LN cells abrogates lymphangiogenesis and lymphatic metastasis *in vivo* in nude mice.

**Results:**

A comparison of expression of cyclo-oxygenase (COX)-2 (a VEGF-C/-D inducer), VEGF-C/-D and their receptors revealed little COX-2 expression by either cells. However, 468LN cells showed differential VEGF-D and α9β1 expression, VEGF-D secretion, proliferative, migratory/invasive capacities, latter functions being stimulated further with VEGF-D. The requirement of α9β1 for native and VEGF-D-stimulated proliferation, migration and Erk activation was demonstrated by treating with α9β1 blocking antibody or knock-down of α9. An autocrine role of VEGF-D in migration was shown by its impairment by silencing VEGF-D and restoration with VEGF-D. 468LN cells and their soluble products stimulated tube formation, migration/invasiveness of HMVEC-dLy cell in a VEGF-D dependent manner as indicated by the loss of stimulation by silencing VEGF-D in 468LN cells. Furthermore, 468LN cells showed α9-dependent stimulation of migration/invasiveness by macrophage products. Finally, capacity for intra-tumoral lymphangiogenesis and lymphatic metastasis in nude mice was completely abrogated by stable knock-down of either VEGF-D or α9 in 468LN cells.

**Conclusion:**

Differential capacity for VEGF-D production and α9β1 integrin expression by 468LN cells jointly contributed to their lymphatic metastatic phenotype.

## Introduction

Metastasis by the lymphatic route, often the first mode of spread of human breast cancer, negatively impacts patient survival [1]. However, the underlying mechanisms remain poorly understood. Vascular endothelial growth factors (VEGF)-C and -D were shown to stimulate lymphangiogenesis by binding to VEGF receptor (R)-3 expressed by lymphatic endothelial cells [2], [3]. Tumoral expression of both these growth factors has been implicated in lymphatic metastasis in human breast cancer [4]–[6]. Earlier we have shown that overexpression of cyclo-oxygenase (COX)-2, an inflammation-associated enzyme, upregulated VEGF-C expression and secretion by human breast cancer cells, thereby promoting lymphangiogenesis in situ and lymphatic metastasis [7], [8]. Additionally, tumor derived VEGF-C served as an autocrine stimulus for breast cancer cell migration by binding to a diverse group of VEGF-C receptors, thus promoting their metastatic ability by both vascular and lymphatic routes [9]. Many studies have utilized metastatic variants of breast cancer cell lines to understand multiple cellular steps and molecular mechanisms involved in metastasis. MDA-MB-468LN cell line (henceforth called 468LN cells) was derived as a lymph node metastasizing variant of the MDA-MB-468GFP human breast adenocarcinoma cell line (henceforth called 468GFP cells) in the laboratory of one of the authors (AFC). 468LN cells produced extensive lymph node metastasis following orthotopic injection in nude mice [10]. They exhibited increased malignant phenotype *in vitro*, and overexpression of α9β1 integrin and its ligand osteopontin, compared with the parental line. The profound *in vitro* and *in vivo* phenotypic and molecular differences within this pair of cell lines presented a unique model for elucidating mechanisms in lymph node metastasis of breast cancer. The integrin α9β1 is a receptor for extracellular matrix (ECM) proteins such as tenascin and osteopontin and for the two lymphangiogenic growth factors VEGF-C and VEGF-D [11]. Overexpression of both osteopontin, a metastasis-associated molecule [12]–[14], and its receptor α9β1 may provide the cells with a metastatic advantage. Subsequent studies revealed some epigenetic signatures of metastasis [15], distinctive chromosomal aberrations [16] and differential expression of genes associated with a ‘cancer stem cell-like’ phenotype [17] in 468LN cells as compared to 468GFP cells. However, precise molecular mechanisms responsible for the enhanced lymphatic metastatic ability of these cells remained unclear. Present study was designed to achieve this goal, demonstrating for the first time that this ability depended on the differential expression of α9β1 and its lymphangiogenic ligand VEGF-D by 468LN cells.

*In vitro* assays for cellular migration and invasion by cancer cells, and tube formation and migration by endothelial cells, have provided an opportunity to elucidate autocrine and paracrine pathways utilized by cancer cells and endothelial cells in promoting angiogenesis [18]–[20] and lymphangiogenesis [21]–[24], which support metastasis by the vascular and lymphatic routes. Current study explored whether a differential expression of VEGF-C or VEGF-D and one or more of the cognate VEGF-C/D receptors by 468LN cells may contribute to their differential capacity for lymphatic metastasis by equipping the cells with a dual advantage: an increase in VEGF-C or -D mediated autocrine motility by utilizing the cognate receptors; and an increased ability for inducing lymphangiogenesis in situ by VEGF-C or -D production and thereby lymphatic metastasis *in vivo*. This hypothesis was tested *in vitro* and *in vivo*. Initial *in vitro* studies compared the expression levels of COX-2 (a VEGF-C/D upregulating enzyme), VEGF-C and -D, and VEGF receptors (VEGF-R-2, R-3, α9 integrin) and migratory/ invasive/proliferative functions in the pair of cell lines (468GFP, 468LN). Since these studies excluded the roles of COX-2 or VEGF-C but validated differential expression of α9 integrin and VEGF-D as well as differential migratory/invasive capacities of 468LN cells, additional *in vitro* studies tested (a) the possible dependence of 468LN cell migration/ invasion and migration associated signaling on α9β1 integrin and its ligand VEGF-C or -D produced by cancer cells themselves or by macrophages; (b) whether 468LN cells or their soluble products stimulate tube formation (lymphangiogenesis *in vitro*) and migration/invasiveness of primary human lymphatic microvascular endothelial cells (HMVEC-dLy) in a VEGF-D dependent manner. *In vivo* studies in nude mice used implants of wild type and α9 or VEGF-D silenced 468LN cells to compare tumor growth, tumor-induced lymphangiogenesis, angiogenesis and metastasis to tumor-draining lymph nodes. We conclude from our results that a differential capacity for VEGF-D production and α9β1 integrin expression by 468LN cells provide an autocrine stimulus for migration/invasiveness and a paracrine/juxtacrine stimulus for lymphangiogenesis, which jointly contributed to their profoundly higher ability to metastasize by the lymphatic route.

## Results

### Differential migratory, invasive, proliferative and VEGF-D producing capacity of 486LN cells, as compared to 468GFP cells

**Figure 1:**
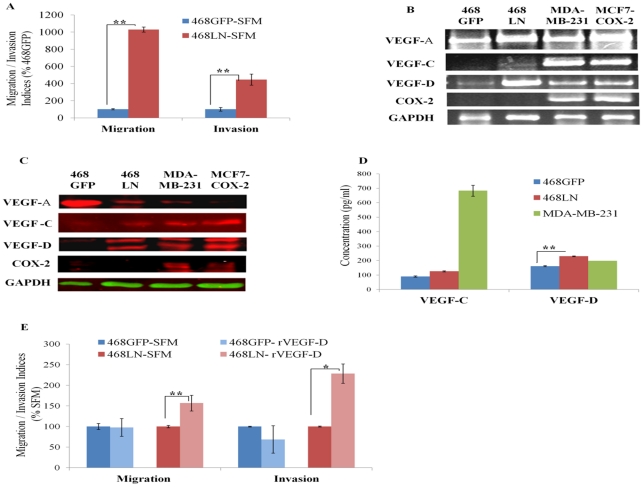
Differential migratory, invasive, and VEGF-D producing capacity of 486LN cells, as compared to 468GFP cells: (**A**) Compared to 468GFP cells, 468LN cells were significantly more migratory and invasive. (**B, C**) 468LN cells expressed significantly higher level of VEGF-D mRNA measured with semi-quantitative RT-PCR (**B**) and total protein measured with western blot (**C**), compared to 468GFP cells. (MDA-MB-231 and MCF7-COX-2 cells served as positive controls for COX-2 and VEGF-C/D). (**D**) 468LN cells secreted significantly higher levels of VEGF-D but not VEGF-C, in comparison to 468GFP cells as measured by ELISA in cell supernatants; MDA-MB-231 cells served as positive controls for both VEGF-C and VEGF-D. (**E**) Exogenous rVEGF-D (2.5 ng/ml) increased both migration and invasion of 468LN, but not 468GFP cells. Migration/ invasion indices were normalized relative to 468GFP in SFM. Migration and invasion of 468GFP-SFM in Fig E was performed separately from the data in Fig A. All bars represent mean (n = 4) +/− S.E, *, P< 0.05; **, P< 0.01.

In earlier studies 468LN cells were shown to have a higher proliferative capacity than the parental 468GFP cells [10]. Here we have confirmed this finding (Fig S1A), and in addition showed that 468LN cells are more migratory and invasive (Quantification: Fig 1A; Image: Fig S1B). To investigate the possible mechanism(s) underlying this differential behaviour, we compared the expression levels of COX-2, VEGF-A, -C or -D (by RT-PCR, western blots) in the two cell lines, because COX-2 was identified in our earlier studies as a migration promoting [7], and VEGF-C upregulating [9] enzyme. Results (expression of mRNA in Fig 1B, S1C and protein in 1C) revealed little or no COX-2 expression by either cell line, but higher levels of VEGF-D expression by 468LN cells. Other human breast cancer cell lines: MDA-MB-231 [7] and MCF7-COX-2 (M Majumder 2011, unpublished) served as positive controls for VEGF-A, -C, -D and COX-2. 468LN cells secreted significantly higher levels of VEGF-D but not VEGF-C (Fig 1D) revealed with ELISA. The migration and invasion of 468LN cells but not 468GFP cells was further stimulated in the presence of exogenous recombinant VEGF-D (rVEGF-D) (2.5 ng/ml) (Quantification: Fig 1E; Image: Fig S1D).

### Reduction of migration/invasiveness and proliferation of 468LN cells by knocking down VEGF-D production

**Figure 2:**
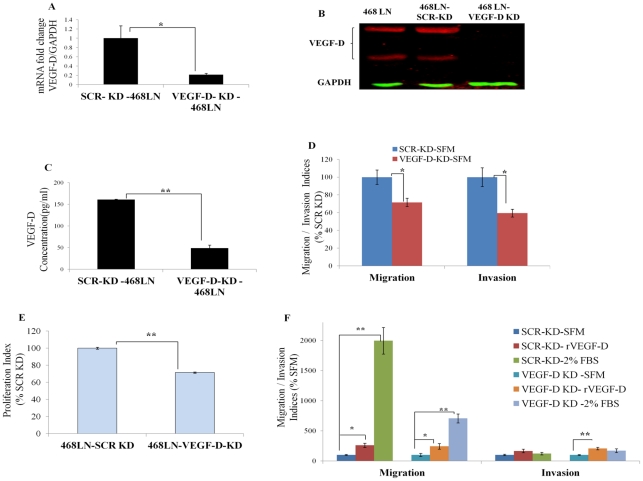
Reduction of migration and invasion of 468LN cells by knocking down VEGF-D production: (**A–C**) Levels of VEGF-D knock down in 468LN cells was confirmed by three different methods, **(A)** qRT-PCR, (**B**) western blot and (**C**) ELISA. After VEGF-D knock down (KD) in 468LN cells, (**D**) migration, invasion and (**E**) proliferation significantly dropped as compared to scrambled knock down (SCR-KD) cells. (**F**) Addition of exogenous rVEGF-D or FBS increased both migration and invasion of VEGF-D knocked down 468LN cells. Bars represent mean (n = 4 in all cases except **C**, n = 6) ± SE, *,P<0.05; **,P<0.01.

We investigated whether siRNA-mediated knocking down of VEGF-D could reduce migratory/invasive/proliferative capacity of 468LN cells. The efficiency of knock down was tested at three different levels, with qRT-PCR (Fig 2A), western blot (Fig 2B) and ELISA (Fig 2C). All these parameters showed a significant drop when cells were transfected with VEGF-D siRNA as compared with scrambled siRNA (Fig 2: A, B and C). Concomitantly, both migratory and invasive capacities of 468LN cells were significantly reduced by silencing VEGF-D (Quantification: Fig 2D; Image: Fig S2) and restored by addition of exogenous rVEGF-D (2.5 ng/ml) (Quantification: Fig 2F; Image: Fig S2). These results strongly support the autocrine migration/invasion promoting role of endogenous VEGF-D. Similarly, a significant drop in proliferation resulting from VEGF-D knockdown of 468LN cells (Fig 2E) indicated additional proliferation-promoting role of endogenous VEGF-D.

### Expression of VEGF-D-binding receptors by 468LN cells

**Figure 3:**
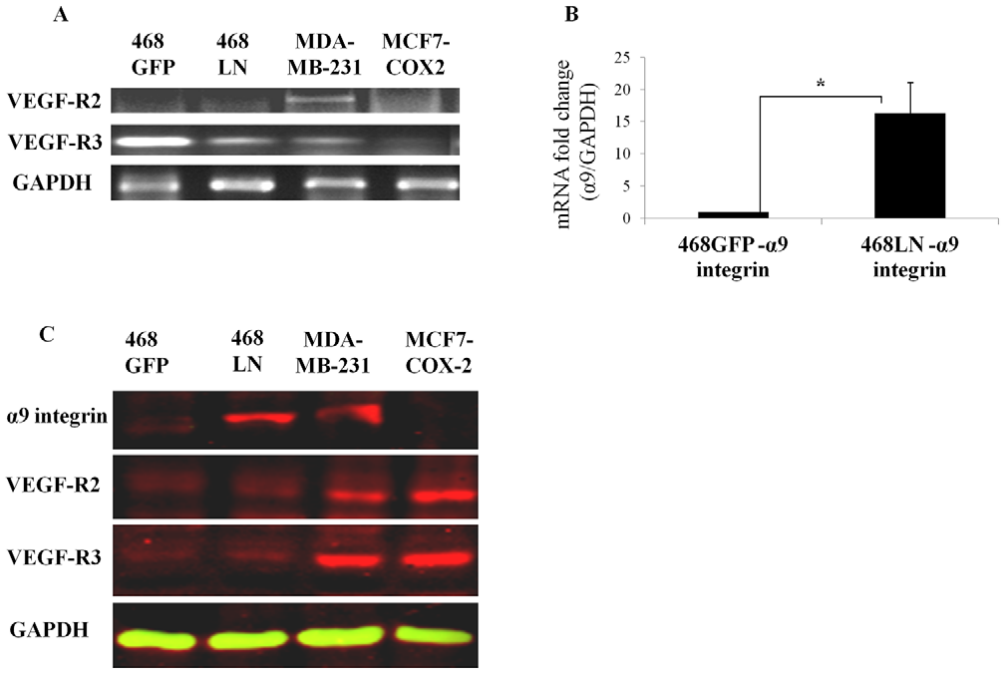
Expression of VEGF-D-binding receptors by 468LN cells: (**A**) Expression of VEGF-R2 and R3 mRNA examined with semi quantitative RT-PCR, and (**B**) α9 integrin mRNA measured with qRT-PCR. (**C**) Confirmation of VEGF-R2, VEGF-R3, and α9 integrin expression at protein levels by western blot. MDA-MB-231 and MCF7-COX-2 cells were used as positive controls primarily for VEGF-R2 and VEGF-R3 expression. Differentially high α9 integrin expression by 468 LN cells was evident. Bars represent mean (n = 4) ± SE, *,P<0.05.

The biological actions of VEGF-D can be mediated theoretically through two tyrosine kinase receptors VEGFR-2 [25] and VEGFR-3 [26] and α9β1 integrin [11]. To determine which of these receptors was responsible for the autocrine effects of VEGF-D, we analysed both mRNA and protein expression of these receptors in 468GFP and 468LN cells, along with other human breast cancer cell lines: MDA-MB-231 [9] and MCF7-COX-2 (M Majumder 2011, unpublished) served as positive controls for VEGF-R2 and VEGF-R3 expression. As reported earlier [9], [10], 468LN cells expressed high levels of α9β1 integrin. Of all the receptors investigated, differential expression of α9 in 468LN cells was most significant at both mRNA (Fig 3: A, B) and protein (Fig 3C) levels.

### Blockade of interaction of α9β1 with its ligand VEGF-D hinders migratory and invasive functions of 468LN cells and α9-associated signaling

**Figure 4:**
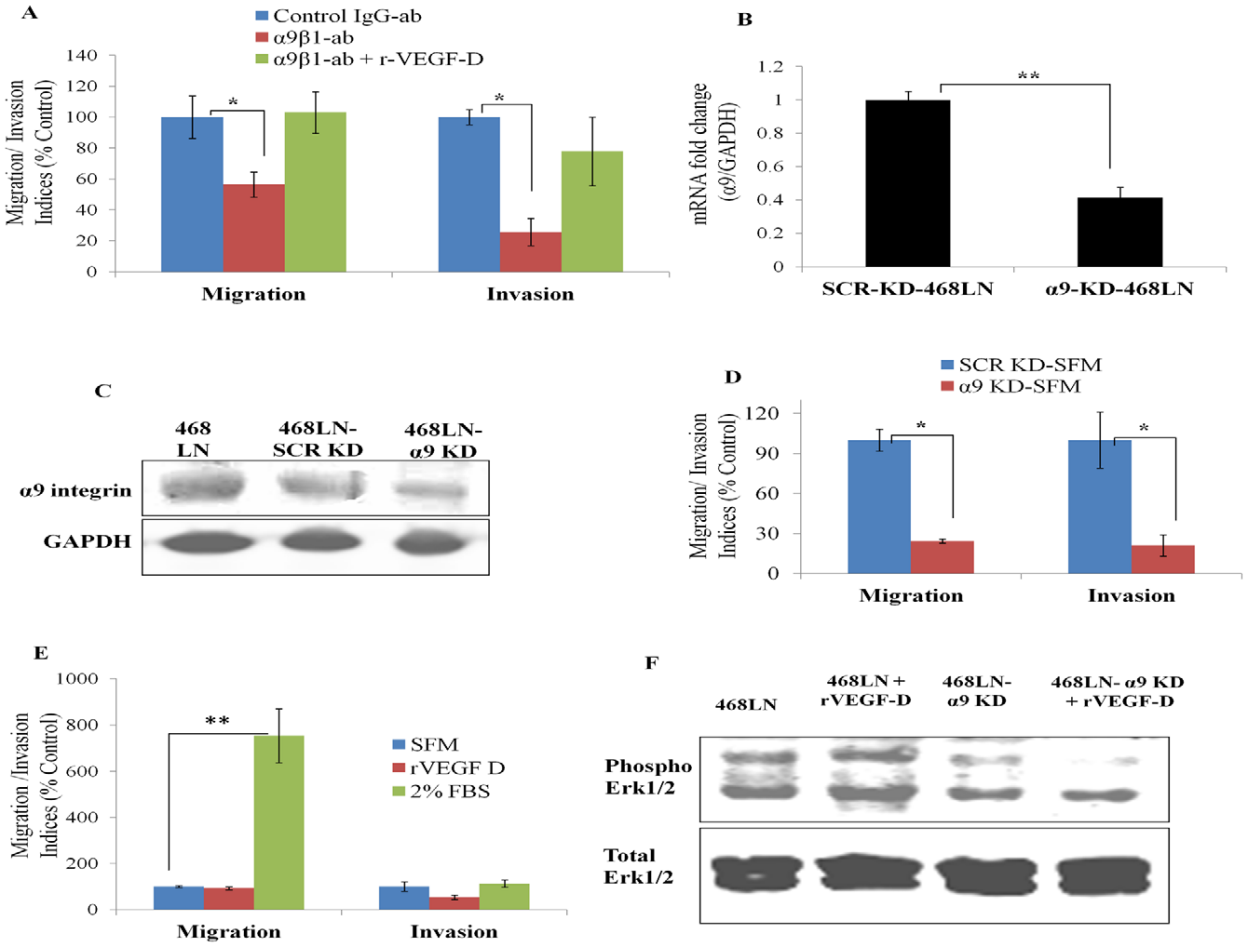
Blockade of α9β1/VEGF-D interaction hinders autocrine migratory/invasive functions of 468LN cells and α9-associated signaling: (**A**) A strong inhibition of both migration and invasion indices of 468LN cells, noted with α9β1 blocking antibody (5 µg/ml). This inhibition could not be reversed by addition of rVEGF-D. Data were normalized to control IgG for α9β1 blocking antibody (ab) and to α9β1 blocking antibody treated cells for rVEGF-D. Knock down of α9 was validated with qRT-PCR (**B**) and western blot (**C**). (**D**) Knock down of α9 integrin (α9-KD) significantly compromised both migration and invasion of 468LN cells compared to scrambled knock down (SCR-KD) cells. (E) Following α9-KD, but not SCR-KD, migratory and invasive functions could be stimulated with FBS, but not with rVEGF-D. Data presented as the mean (n = 4 except invasion data in D, n = 6) ± SE. *, P<0.05; **,P<0.01. (F) Phospho Erk1/2 was measured using western blots in wild type and α9-silenced, serum starved (24 h) 468LN cells in the presence or absence of rVEGF-D (3 µg/ml) at 0–60 min as compared to total Erk1/2. Significant constitutive Erk1/2 activity noted in the absence of rVEGF-D was stimulated further in the presence of rVEGF-D at 15–45 min (peak shown at 45 min). Knock down of α9 integrin resulted in suppressed Erk1/2 activity in both cases.

**A**utocrine **R**egulation: First, we tested whether a mouse monoclonal α9β1 blocking antibody (1 and 5 µg/ml, added to cells 0.5 h prior to and during these assays) could impair 468LN cell migration and invasiveness. This antibody had been shown to inhibit VEGF-C/-D induced migration of α9 transfected mouse embryonic fibroblasts and primary adult human dermal microvascular endothelial cells [11]. Both migration and invasion (Fig 4A) of 468LN but not 468GFP cells (data not shown) were significantly inhibited in the presence of the α9β1 blocking antibody (5 µg/ml) as compared to control IgG (5 µg/ml). This inhibition could not be reversed by addition of rVEGF-D (2.5 ng/ml) (Fig 4A). Second, we tested whether silencing α9 integrin gene could impair migration/ invasiveness of 468LN cells. Cells were transfected with α9 siRNA (1 µM) and the following four parameters were measured: α9 mRNA level (Fig 4B), α9 protein level (Fig 4C), cellular migration (Quantification: Fig 4D; Image: Fig S3), and invasion (Fig 4D). We observed a significant drop in each of the parameters, when α9 integrin transfected cells were compared with scrambled siRNA treated cells. This inhibition could not be reversed by addition of rVEGF-D (Quantification: Fig 4E; Image: Fig S3), validating that α9 was the dominant receptor for VEGF-D action in these cells. Since Erk activation is a key signaling pathway mediated by α9 interaction with VEGF-D [11], we compared Erk phosphorylation in wild type and α9-silenced 468LN cells in the absence or presence of VEGF-D. The phosphorylation level was significantly suppressed by α9 knockdown which could not be restored with rVEGF-D (Fig 4F). These results taken together, affirm the autocrine role of VEGF-D interacting with α9β1 integrin in promoting migration and invasion of 468LN cells.

**Figure 5:**
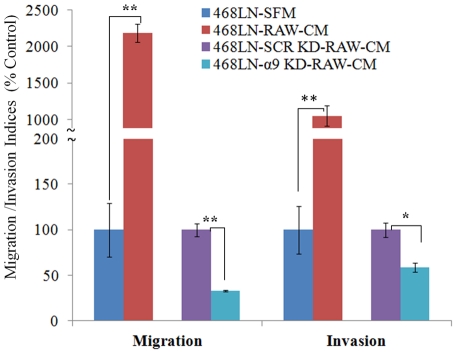
Paracrine migration/invasion promoting role of macrophage derived factors on 468LN cells is largely α9 dependent: Conditioned medium (CM) of RAW 264.7 macrophage cells strongly stimulated both migration and invasiveness of 468LN cells. This stimulation was significantly abrogated by knocking down the α9 integrin (α9-KD) in 468LN cells, as compared to scrambled siRNA knock down (SCR-KD) cells. The knockdown of α9 integrin was done as in Fig 4. Data presented as the mean (n = 4) ± SE. *, P<0.05; **,P<0.01.

**P**aracrine **R**egulation: Tumor-associated macrophages have been reported to stimulate tumor progression and metastasis by producing soluble factors including VEGF-C and D [27]. VEGF-C and -D production by macrophages also contributes to lymphangiogenesis during inflammation [28]. Thus a paracrine role of VEGF-C or -D producing tumor-associated macrophages in situ in promoting α9-dependent motility or invasiveness of 468LN may also contribute to their lymphatic metastatic phenotype. We tested this possibility by utilizing a VEGF-C and -D producing surrogate macrophage cell line RAW 264.7. We observed markedly increased migration and invasiveness of 468LN cells when macrophage cell-conditioned medium was placed in the bottom chamber. This increase was significantly abrogated by siRNA mediated knockdown of α9 in 468LN cells in comparison with scrambled siRNA treated cells (Quantification: Fig 5; Image: Fig S4).

### VEGF-D production by 468LN cells increases capillary-like tube formation by HMVEC-dLy cells via paracrine and juxtacrine mechanisms

**Figure 6:**
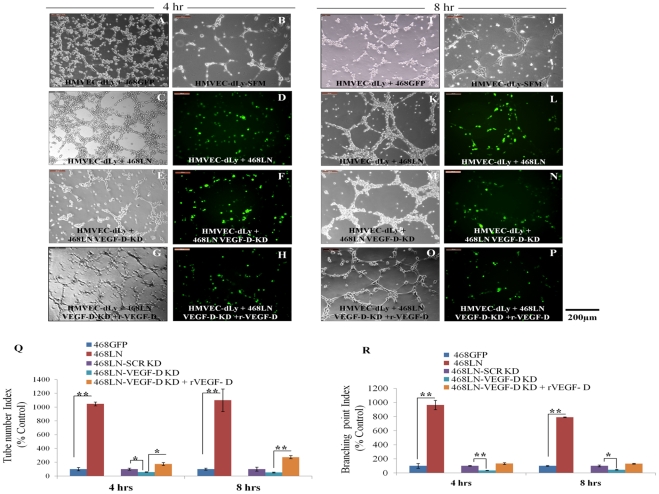
VEGF-D production by 468LN cells increased capillary-like tube formation of HMVEC-dLy cells: 468GFP cells when co-cultured with HMVEC-dLy cells formed sparse and incomplete tube like structure at either 4 h (**A**) or 8 h (**T**), which were not different from HMVEC-dLy cells alone at both 4 h (**B**) and 8h (**J**) when seeded on growth factor reduced Matrigel in serum free medium (SFM). When 468LN cells were co-cultured with HMVEC-dLy cells, they showed enhanced formation of complete tubes as early as 4 h (**C**) and 8 h (**K**). (**D, L**) 468LN cells express GFP, so that it was evident from the images of tubes in co-culture, that both cancer cells and HMVEC-dLy cells aligned together in forming tubes, indicating cell-cell interaction or collaboration in aligned tube formation. (**E, M**) To test whether 468LN cell-derived VEGF-D contributed to tube formation by HMVEC-dLy cells, the same experiments were done after silencing the endogenous VEGF-D of 468LN cells. In this case, tube formation in co-cultured cells were reduced but not completely abolished. (**G, O**) Cells regained their tube forming capacity, when rVEGF-D (2.5 ng/ml) was added to the medium. (**F, H, N, P**) Again the GFP marker in 468LN cells confirms that these tubes were not exclusive to HMVEC-dLy. Quantitative data presented as (**Q**) tube number and (**R**) branching point indices. Data represent (n = 3) ± SEM. *,P<0.05; **,P<0.01.

*In vitro* lymphangiogenesis assays utilize the formation of three-dimensional tube or vessel like structures by lymphatic endothelial cells [29]–[31]. Cancer cells can also form pseudo-vascular tubes on Matrigel, the phenomenon known as vascular mimicry [32], [33], the temporal kinetics of tube formation differing from cell to cell [34], [35]. Upon plating serum-starved cells on growth factor reduced Matrigel we noted that both 468LN and HMVEC-dLy cells could individually form tube like structures, but with different kinetics. HMVEC-dLy cells formed tubes as early as 8 h (Fig 6: B, J) whereas 468LN cells required 12 h or more to show the earliest signs of tubulogenesis, at which time no tubulogenesis was evident with 468GFP cells (not shown). We tested the paracrine role of 468LN cells for lymphangiogenesis via VEGF-D production, measured with tube formation by HMVEC-dLy cells in the presence of 468LN conditioned medium. In addition, possible juxtacrine effects (defined as the local effects of a cell membrane bound molecule on another cell nearby) of 468LN cells on tube formation by HMVEC-dLy cells were tested in co-cultures of cancer cells and HMVEC-dLy cells.

**C**o-culture: When HMVEC-dLy cells were co-cultured with cancer cells on Matrigel at 1∶1 cell ratio, tubes formed as early as 4 h with 468LN cells (Fig 6: C, K) and the number of tubes and branching points were significantly higher compared to co-culture with 468GFP cells at both 4 h and 8 h (Image: Fig 6 A, I; Quantification: Q, R) or those in HMVEC-dLy cells cultured alone (Fig 6: B, J). From images of tubes in co-culture, it was evident that both lymphatic endothelial and GFP-marked cancer cells (green) aligned together in forming tubes (Fig 6: D, L), indicating cell-cell interaction or collaboration in tube formation. When VEGF-D was knocked down in 468LN cells, tube formation in co-cultured cells were reduced but not completely abolished (Fig 6: E, M) in comparison with those in co-cultures including scrambled siRNA treated tumor cells (Image not shown; Quantification: Fig 6: Q, R). Cells regained their tube forming capacity when rVEGF-D (2.5 ng/ml) was added to the medium (Image: Fig 6 G, O and Quantification: Q, R). Again the GFP marker in 468LN cells confirms that these tubes were not exclusive to HMVEC-dLy cells (Fig 6: D, F, H, L, N, P). We speculate that the VEGF-D mediated enhancement of lymphangiogenesis in co-cultures represents a juxtacrine effect of cancer cells. In other words, binding of tumor-derived VEGF-D to α9 integrin receptor on cancer cells leaves its VEGF-R3 binding site unoccupied, so that the same VEGF-D molecule can also bind to VEGF-R3 expressed by HMVEC-dLy cells to mediate the lymphangiogenic stimulus.

**Figure 7:**
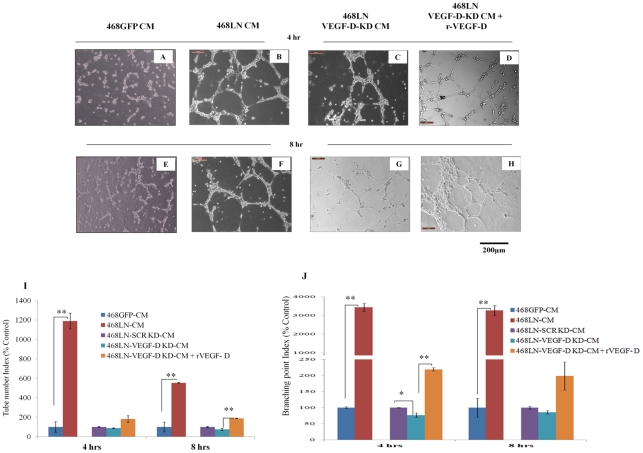
468LN cell conditioned medium increases capillary-like tube formation by HMVEC-dLy cells: (**A, E**) HMVEC-dLy cells could hardly form any tube like structure with addition of conditioned medium (CM) from 468GFP cells at (**A**) 4 h and (**E**) 8 h. (**B, F**) HMVEC-dLy cells could form complete tubes as early as at (**B**) 4 h and (**F**) 8 h with 468LN CM. (**C, G**) VEGF-D knock down (KD) in 468LN cells, resulted in reduction in tube formation at both time points. (**D, H**) Again, addition of rVEGF-D in the CM of VEGF-D-KD 468LN cells stimulated tube formation. (**I, J**) Quantitative data for above observations are presented as (**I**) tube number index and (**J**) branching point index showing significant increase in tube and branch formation in presence of 468LN cell CM, but not 468GFP CM. Moreover VEGF-D KD in 468LN cells could block this function and HMVEC-dLy cells regained this function when rVEGF-D was added. Data represent mean (n = 3) ± SE. *,P<0.05; **,P<0.01.

**4**68LN **C**onditioned **M**edium: Next, we investigated the paracrine effect of VEGF-D produced by 468LN cells on tube formation by HMVEC-dLy cells. Briefly, 468LN and 468GFP cells (used as control) were grown for 24 h in regular medium, and serum-starved for another 12 h to collect the conditioned medium. This conditioned medium was added to HMVEC-dLy cells and plated on Matrigel. As observed previously in co-culture, HMVEC-dLy cells formed tubes with an accelerated tempo at both the time points 4 h and 8 h (Image: Fig 7 B, F and Quantification: I, J) in the presence of 468LN CM compared to 468GFP CM, validated by the number of tubes and branching points (Image: Fig 7 A, E and Quantification: I, J). To test the role of VEGF-D as a secreted product of 468LN cells in the induction of tube formation by HMVEC-dLy cells, the same experiments as above were duplicated after siRNA mediated VEGF-D knock down of 468LN cells. Tube formation at both the time points, with CM from VEGF-D silenced 468LN cells, was significantly reduced but not completely abolished when compared to scrambled siRNA knock down (Image: Fig 7 C, G and Quantification: Fig I, J). This can be explained by the presence of other stimulants present in growth factor reduced Matrigel or other products secreted by the cells. Again, addition of exogenous rVEGF-D (5 ng/ml) promoted tube formation (Image: Fig 7 D, H and Quantification: I, J). The above results taken together confirmed the paracrine and juxtacrine roles of 468LN cells on lymphangiogenesis resulting from VEGF-D production.

### VEGF-D secreted by 468LN cells promotes migration and invasion by HMVEC-dLy cells

**Figure 8:**
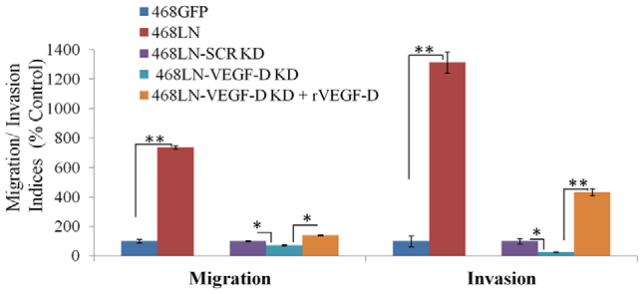
VEGF-D secreted by 468LN cells promotes migration and invasion by HMVEC-dLy cells: Significant stimulation of both migration and invasion of HMVEC-dLy cells occurred in the presence of 468LN conditioned medium (CM), as compared to 468GFP CM. This stimulation was significantly attenuated by VEGF-D knock down (KD) in 468 LN, which could be restored with exogenous rVEGF-D. For VEGF-D knock down, data were normalised to scrambled siRNA (SCR-KD), and for rVEGF-D stimulation, data were normalised to VEGF-D knock down. Data represent mean (n = 4) ± SE. *,P<0.05; **,P<0.01.

Since lymphatic endothelial cell migration and invasion of basement membranes are essential steps for lymphangiogenesis *in vivo* [36], we examined the paracrine effect of VEGF-D secreted by 468LN cells in promotion of migratory and invasive functions of HMVEC-dLy cells. 468LN cells (added in the bottom chamber) were found to stimulate migration as well as invasion (Image: Fig S5; Quantification: Fig 8) of HMVEC-dLy cells compared to 468GFP cells added to the bottom chamber. When similar experiments were done using VEGF-D-silenced 468LN cells in the bottom chamber following transfection with VEGF-D siRNA (5 µM), the migration and invasiveness of HMVEC-dLy cells was lower than those observed with un-manipulated 468LN cells (Image: Fig S5; Quantification: Fig 8). More interestingly, this reduction could be abrogated with addition of exogenous r-VEGF-D to the VEGF-D silenced 468LN cells (Image: Fig S5; Quantification: Fig 8). These results further define the cellular steps underlying the paracrine role of 468LN cell-derived VEGF-D in promoting lymphangiogenesis.

### VEGF-D induced migration of HMVEC-dLy cells is Erk dependent

**Figure 9:**
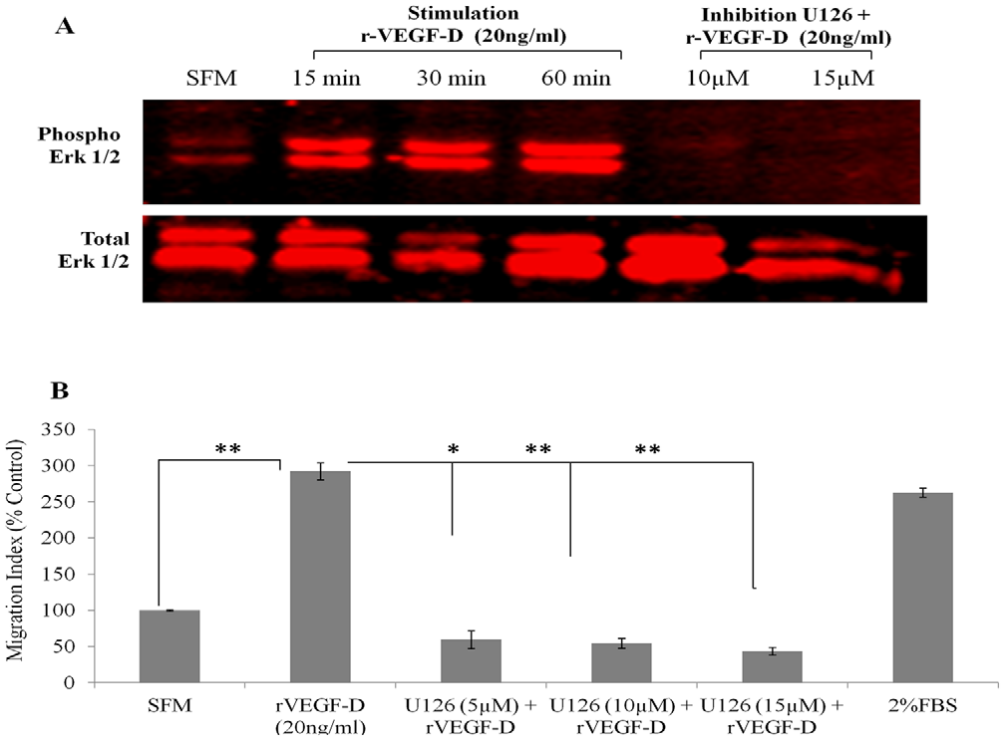
VEGF-D induced migration of HMVEC-dLy cells is Erk dependent: (**A**) Cells were serum starved for 24 h before stimulation with r-VEGF-D (20 ng/ml) for varying lengths of time (0, 15, 30 and 60 minutes) or pre-treated with varying concentrations of Erk inhibitor U0126 (0, 5, 10 and 15 µM) for one hour and further treated with 20 ng/ml r-VEGF-D for 30 minutes. Cell lysates were prepared and analyzed by western blot for Erk1/2 activity. VEGF-D stimulated Erk activation in HMVEC-dLy cells and this stimulation was prevented by pre-treatment with U126. (**B**) Cell migration was quantified by transwell migration assays; VEGF-D stimulated migration was inhibited with U126 (5–15 µM). 2% FBS served as a positive control. Migration data were represented as a mean ± S.E. for three independent experiments (n = 3). For recombinant VEGF-D stimulation, data were normalised to SFM and for Erk inhibition data were normalised to r-VEGF-D stimulation.

Since mitogen-activated protein kinases (MAPKs) have been implicated in VEGF-stimulated migration in a variety of cell types [37], [38], HMVEC-dLy cells were stimulated with 20 ng/ml r-VEGF-D for 15–60 minutes and processed for western blots. Phosphorylation of Erk1/2 was induced by recombinant VEGF-D (r-VEGF-D), reaching maximal activation at 60 minutes (Fig 9A). Pretreatment with Erk1/2 inhibitor U0126 (10, 15 µM) for 60 minutes completely blocked Erk phosphorylation which could not be restored by addition of r-VEGF-D (Fig 9A). Furthermore, migration stimulation of HMVEC-dLy cells with rVEGF-D was significantly compromised by pretreating the cells with U0126 at a concentration as low as 5 µM (Image: Fig S6; Quantification: Fig 9B).

### Experimental design *in vivo*

We tested the individual contributions of VEGF-D and α9 integrin to the lymphatic metastatic phenotype of 486LN cells *in vivo* by stably knocking down either molecule with shRNA plasmids. *In vivo* experiments were performed with these two stable cell lines named Δα9/468LN and ΔVEGF-D/468LN for α9 integrin and VEGF-D stable knock down respectively. Since control shRNA knock down showed no difference in protein expression when compared to parental 468LN cells (Fig S7), we conducted *in vivo* experiments with parental 468LN cells as positive control for VEGF-D and α9β1 integrin expression. Tumor growth, lymphatic ingrowth into the tumors, intratumoral lymphangiogenesis as well as angiogenesis, and lymphatic metastasis were measured after subcutaneous implantation of tumor cells suspended in growth factor reduced Matrigel. This implantation method allowed ingrowths of capillaries and lymphatics into the developing tumors from pre-existing vasculature. Matrigel implants devoid of tumor cells served as negative controls.

### VEGF-D knock down reduces lymphatic ingrowths into the tumor

**Figure 10:**
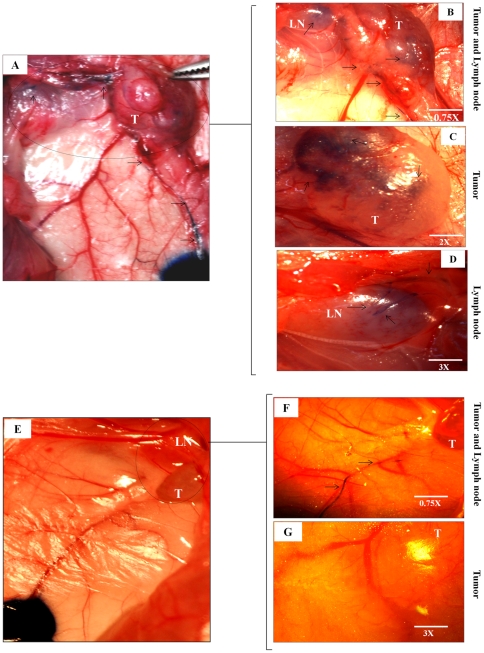
VEGF-D knock down reduced lymphatic ingrowths into the tumor: (**A**) Digital camera captured picture at 5 minutes show the Evans blue dye injection sites and the dye stained lymphatics going towards the 468LN tumor (T). (**B**) Images were captured at 15 minutes using a dissection microscope. These images show lymphatics (arrows) going into the 468LN tumors as well as tumor draining axillary lymph nodes (LN). (**C**) Image of same tumor at a higher magnification (see inset) captured at 20 minutes showing blue dye inside the tumor (T), and (**D**) inside the lymph node (LN) at a higher magnification (see inset). (**E**) In contrast, VEGF-D knock down (ΔVEGF-D/468LN) tumor implants showed very few lymphatics traceable from the injection site captured with a digital camera. (**F**) Picture taken at 15 minutes showing lymphatics alone but very little dye in the tumor. (**G**) Even at 25 minutes no dye-stained lymphatics was visible on the surface or inside the tumor at a higher magnification (see inset).

Evans Blue dye injection sites were approximately 2 cm apart from the tumor implants, allowing a visual tracing of lymphatics into both 468LN and ΔVEGF-D/468LN tumors (Fig 10: A, E). Significant infiltration of blue dye in 468LN tumor (Fig 10: B, C) and tumor-draining lymph node (Fig 10: B, D) was observed at 10–15 minutes post injection. In contrast, no dye was visible in ΔVEGF-D/468LN tumors even at 25 minutes (Fig 10: F, G). In ΔVEGF-D/468LN implanted mice, it was hard to visualise draining lymph nodes from the surface because of lack of staining with blue dye, so that no image could be presented.

### Knock down of α9 integrin and VEGF-D in 468LN cells abrogates primary tumor growth in nude mice

**Figure 11:**
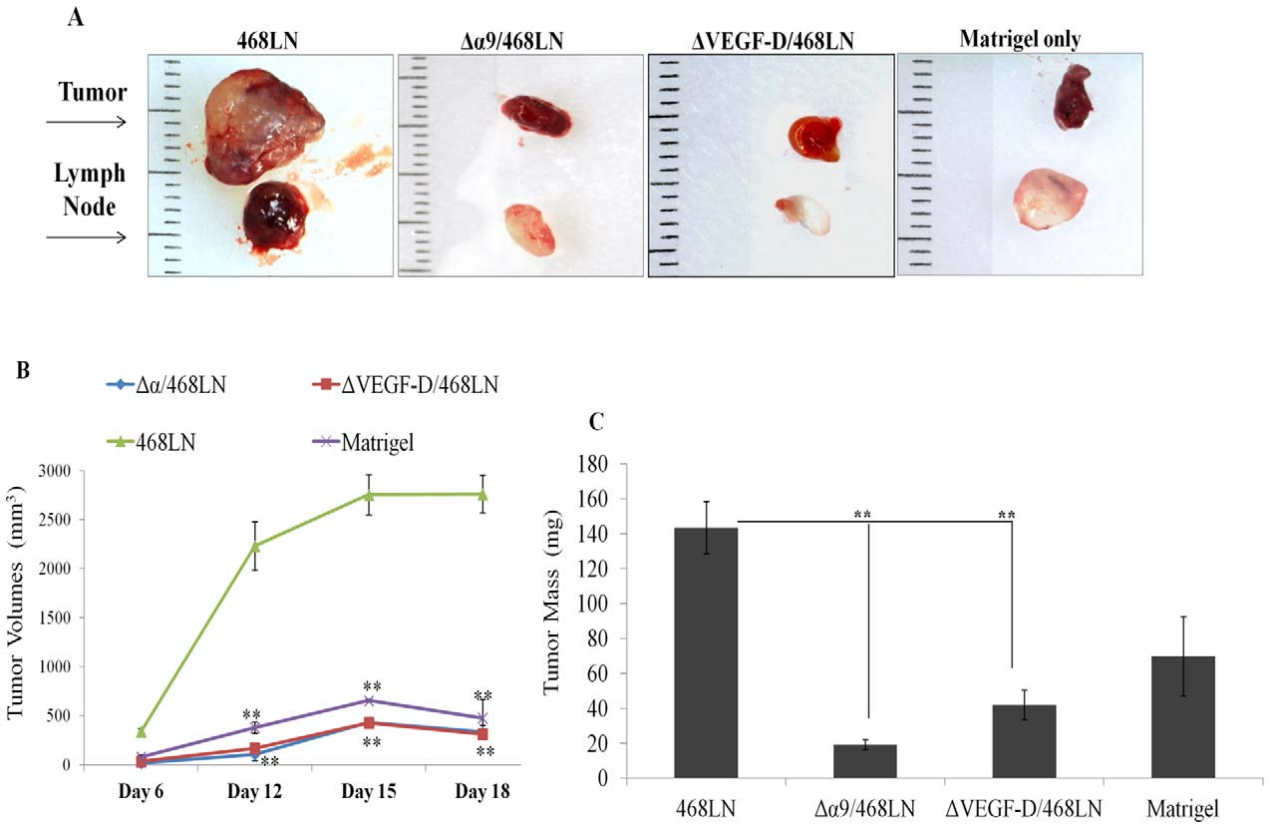
Knock down of α9 integrin and VEGF-D in 468LN cells reduced primary tumor growth in nude mice: (**A**) Representative images of tumors and Matrigel alone as well as the draining lymph nodes on day 20 (a scale in mm shown in the background). (**B**) Tumor growth rates determined by volume measured externally were dramatically reduced in both α9 integrin (Δα9/468LN) and VEGF-D knock down (ΔVEGF-D/468LN) tumors (indistinguishable from Matrigel alone). (**C**) Tumors were excised and mean weights of tumors and Matrigel were retrieved on day 20. Weights of 468LN tumors were significantly higher than those of Matrigel alone or Δα9/468LN and ΔVEGF-D/468LN tumors. Data represented as means (n = 16 for tumors and 4 for Matrigel) ± S.E. *p<0.05, **p<0.001.

Implants of Matrigel alone at four locations (two inguinal, two axillary) in one mouse remained unchanged in size (Fig 11A), serving as negative control. Tumor growth rates given by externally measured volumes (Fig 11B) at days 12, 15, 18 and weights taken at sacrifice on day 20 (Fig 11C) were drastically lower (p<0.001) in mice implanted with Δα9/468LN and ΔVEGF-D/468LN cells compared to 468LN cell implanted mice (gross morphology of representative tumors and lymph nodes in Fig 11A). Tumors from Δα9/468LN and ΔVEGF-D/468LN cells implanted mice were indistinguishable (p>0.07) from Matrigel alone, indicating a complete abrogation of growth.

### Metastatic ability to the draining lymph node was abrogated by α9 integrin and VEGF-D knock down in 468LN cells

**Figure 12:**
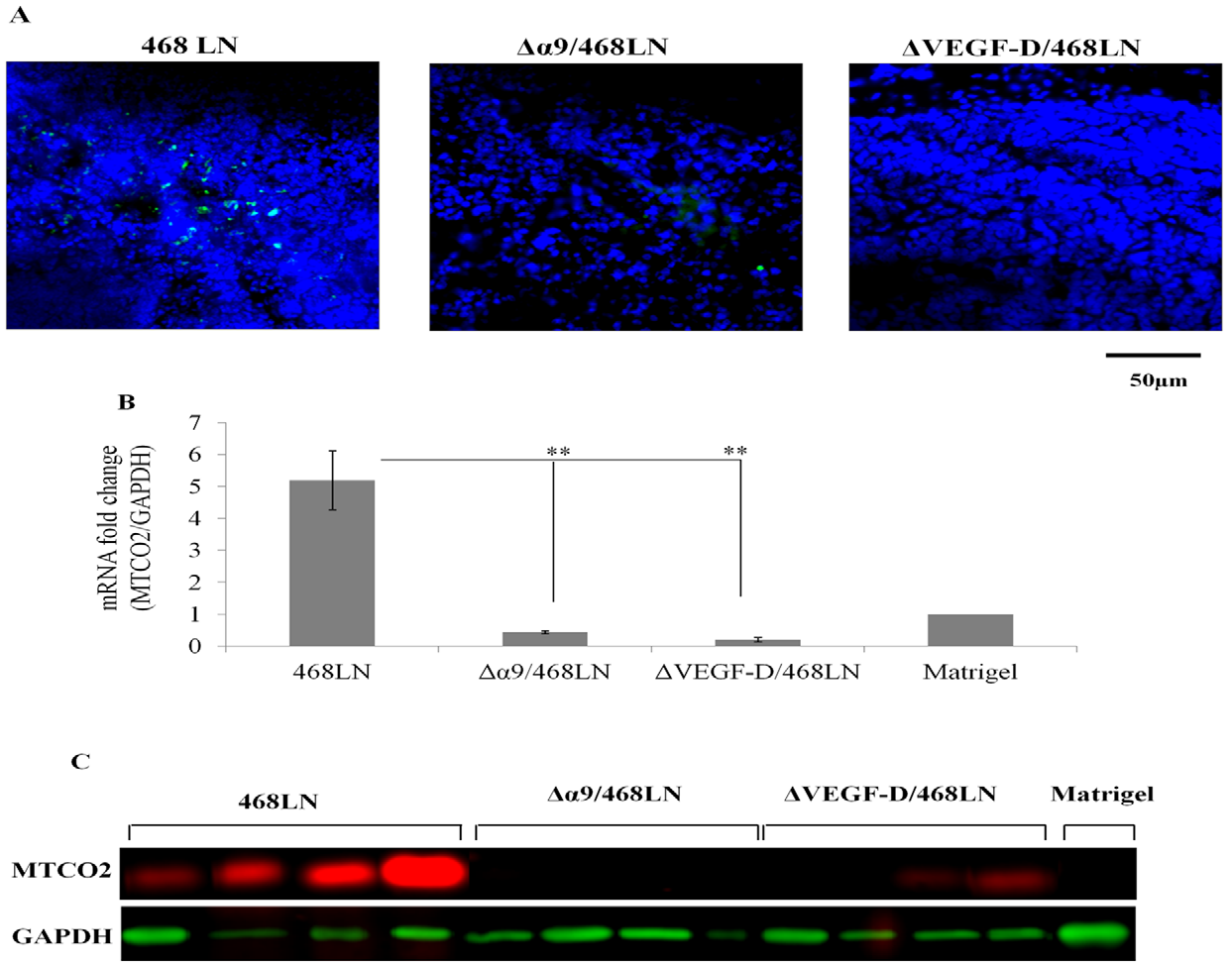
Metastatic spread of tumor cells to the draining lymph nodes is abrogated by α9 integrin and VEGF-D knock down in 468LN cells: (**A**) Metastasis to lymph nodes were validated by the visual localisation of many cancer cells in 468LN tumor draining nodes identified by the GFP-marker and their absence in lymph nodes draining α9 integrin and VEGF-D knock down tumor implants. Nuclei were stained blue by DAPI. (**B, C**) Above conclusions were further validated by measuring the human mitochondrial-specific marker MTCO2 at the mRNA level with qRT-PCR (**B**) and protein level with western blot (**C**) was high in lymph nodes draining 468LN tumors. This process was nearly completely abrogated in both Δα9/468LN and ΔVEGF-D/468LN tumor implants. Data were derived from 16 lymph nodes. Four lymph nodes draining into an individual tumor in a single mouse were pooled to extract RNA and proteins, showing the data in four mice; Matrigel was retrieved from four implants. Data represents mean (n = 4) ± S.E, *p<0.05, *p<0.001.

In frozen sections of lymph nodes draining 468LN tumors, many green 468LN cells (resulting from the GFP marker) were identified by fluorescence microscopy (Fig 12A). In contrast, such cells were hardly visible in the draining lymph nodes from both Δα9/468LN and ΔVEGF-D/468LN tumors, indicating that cancer cells lost their ability to metastasize to the nodes. In an additional approach, we indirectly quantified metastasis using human mitochondrial marker (MTCO2) RNA (Fig 12B) and protein (Fig 12C) expression levels in lymph nodes. Both qRT-PCR and western blots confirmed the abundance of human cancer cell related marker in nodes draining 468LN tumors. Based on the standard plot obtained with predetermined numbers of 468LN cells expressing human mitochondrial marker MTCO2 measured with qRT-PCR (Fig S8), we approximated that on the average 2,000 468LN cells metastasized to the individual 468LN tumor draining lymph node. In contrast, they were undetectable (compared to the back ground given by Matrigel implant) in lymph nodes draining Δα9/468LN and ΔVEGF-D/468LN tumors.

### Ability to induce intra-tumoral lymphangiogenesis is abrogated by both VEGF-D and α9 integrin knock down in 468LN cells

#### Indirect Measurements

**Figure 13:**
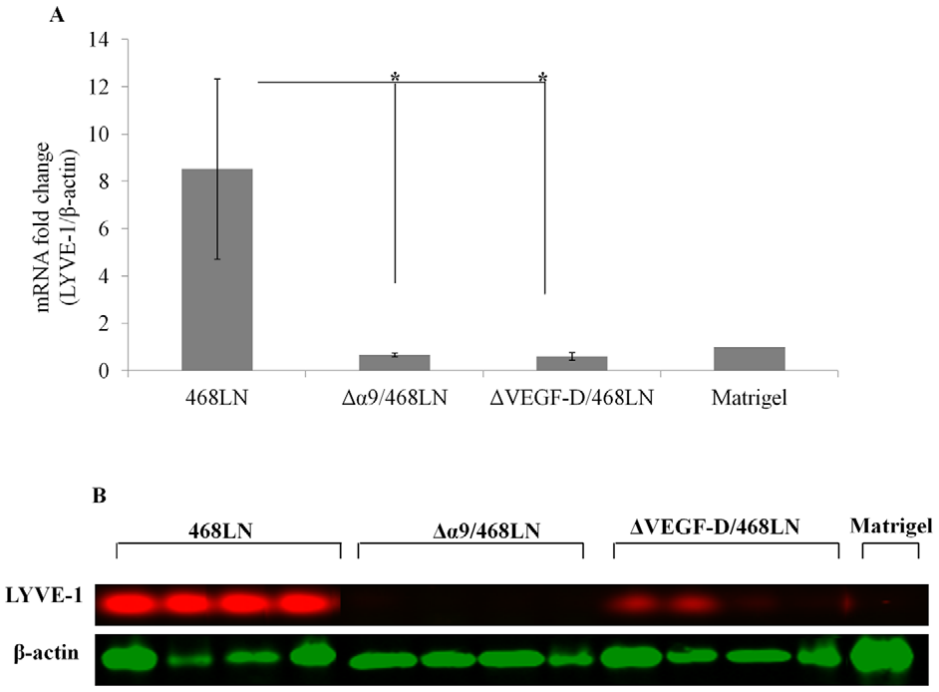
Ability to induce intra-tumoral lymphangiogenesis was abrogated by both VEGF-D and α9 integrin knock down in 468LN cells: (**A, B**) Intra-tumoral lymphangiogenesis was measured indirectly from the expression of murine lymphatic endothelial marker LYVE-1 in tumors at the mRNA level with qRT-PCR (**A**) and protein level with western blot (**B**). Murine LYVE-1 expression was very high in 468LN tumors and this expression was nearly completely abolished in both Δα9/468LN and ΔVEGF-D/468LN tumor implants. Data were derived from 16 tumors. Four tumors in a single mouse were pooled to extract RNA and proteins, showing the data in four mice; Matrigel was retrieved from four implants. Data represents mean (n = 4) ± S.E, *p<0.05, *p<0.001.

To quantify the relative abundance of mouse lymphatics within the human tumor transplants we took advantage of the mouse lymphatic endothelial marker LYVE-1 at protein and mRNA levels. We observed significant reduction in mouse LYVE-1 expression at both mRNA (p<0.05) (Fig 13A) and protein levels (Fig 13B) in both Δα9/468LN and ΔVEGF-D/468LN tumors compared to 468LN tumors.

#### Direct Measurements

**Figure 14:**
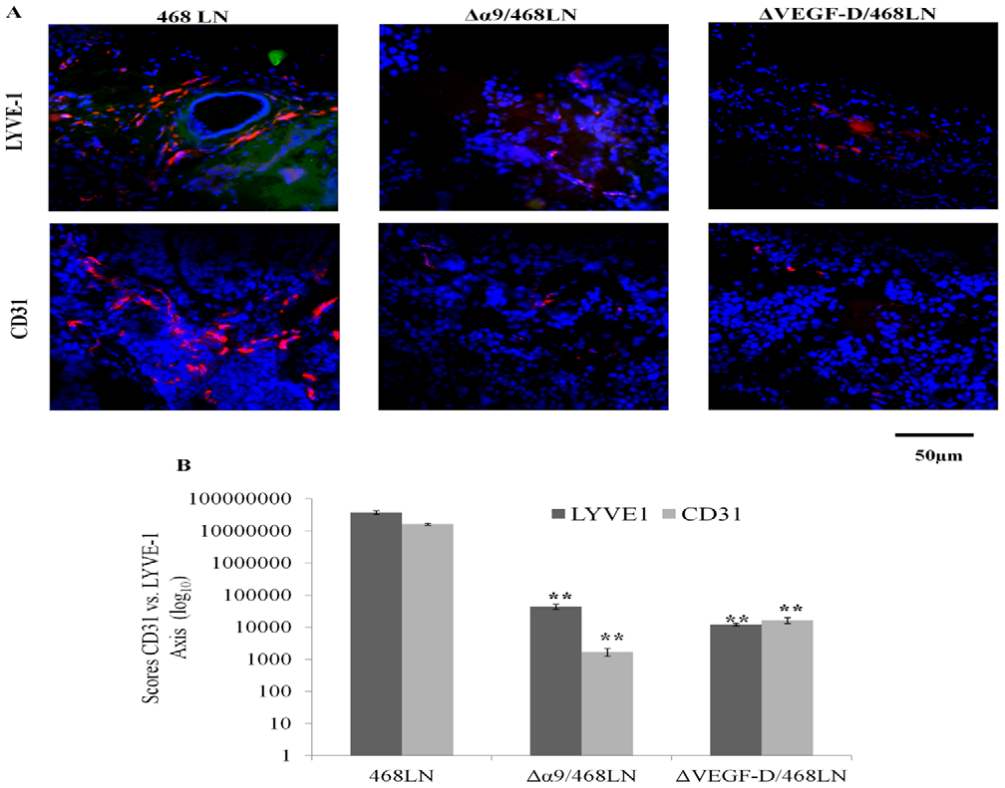
Intra-tumoral lymphangiogenesis (LYVE-1 immunostatining-red) as well as angiogenesis (CD31 immunostaining-red) were abrogated by both α9 integrin and VEGF-D knock down in 468LN cells: (A) Direct measurements of lymphangiogenesis and angiogenesis respectively identified with immunofluorescent labelling for murine LYVE-1 and CD31 markers in serial frozen sections of tumors revealed significantly higher labelling for both markers in 468LN tumors compared to Δα9/468LN and ΔVEGF-D/468LN tumors. Nuclei were stained with DAPI (blue). (B) Fluorescence was quantified as corresponding “hot spot” scores. (n = 16, using the mean of 3 hot spots from each of the 16 tumors per group; Matrigel implants, n = 4) ± S.E, **p<0.001.

In addition to the quantification of LYVE-1 at the mRNA and protein levels, we directly measured the levels of lymphangiogenesis and angiogenesis in serial frozen sections of tumors with immunofluorescent labelling for LYVE-1 (red) and CD31 (red) respectively. CD31 staining for blood vessels (angiogenesis) was also quantified, since it is one parameter that can dictate growth of solid tumors. The immunofluorescence images are representative of “hot spots” within the tumors [39], [40]. High levels of tumor-associated angiogenesis as well as lymphangiogenesis (Fig 14A, and quantified in Fig 14B) occurred in 468LN implanted tumors. These events were nearly completely abrogated in Δα9/468LN and ΔVEGF-D/468LN implants. No measurable angiogenesis or lymphangiogenesis was observed in Matrigel only implants (data not shown).

## Discussion

Since its isolation, the 468LN cell line was shown to be a lymph node metastasizing variant of the MDA-MB-468 human breast adenocarcinoma cell line with increased *in vitro* proliferative capacity and overexpression of α9β1 integrin and osteopontin [10]. While earlier studies revealed multiple mechanisms underlying their metastatic phenotype [15], [16], [17], the precise molecular mechanism(s) responsible for their increased lymphatic metastatic ability remained undefined. In the present study, utilizing multiple *in vitro* and *in vivo* assays, we have dissected the mechanisms underlying the capacity of the cells in promoting lymphangiogenesis, lympho-vascular invasion and lymphatic metastasis. We have excluded the differential role of COX-2 expression in VEGF-C or D upregulation and show that differential overexpression of α9β1 and its ligand VEGF-D by 468LN cells underlie these events. Screening of several human breast cancer cell lines revealed that 468LN cells express the highest level of both markers (Fig S9). However, another cell line, MDA-MB-231, also known for its ability for lymphatic metastasis, expresses both these markers. This observation suggests that these markers may also contribute to lymphatic metastasis in addition to the earlier reported COX-2 mediated upregulation of VEGF-C [7], [8] in this cell line. Since a diversity of *in vitro* derived cell lines may reflect the tumor cell heterogeneity with some degree of clonal dominance in situ in advanced cancers, we suggest that present results are clinically relevant. We show that dual over expression of α9β1 integrin and its ligand VEGF-D by 468LN cells lead to multiple mechanisms in promoting lymphatic metastasis. While VEGF-D production accounted for their robust ability to induce lymphangiogenesis *in vitro* and *in vivo*, its interaction with α9β1 equipped them with an autocrine stimulus for motility and invasiveness, which represent key steps in the process of metastasis by both vascular and lymphatic routes. Furthermore, tumor-derived VEGF-D served both as a paracrine as well as a juxtacrine stimulus for tumor-induced lymphangiogenesis. Finally, our *in vivo* studies in nude mice confirmed the obligatory roles of both α9 integrin receptor and its ligand VEGF-D in promoting tumor-associated lymphangiogenesis and lymphatic metastasis. Present study, to our knowledge, is the first report of a dual role of this receptor-ligand interaction in human breast cancer, in events associated tumor growth and metastasis, in particular, with a lymphatic metastatic phenotype.

**Figure 15:**
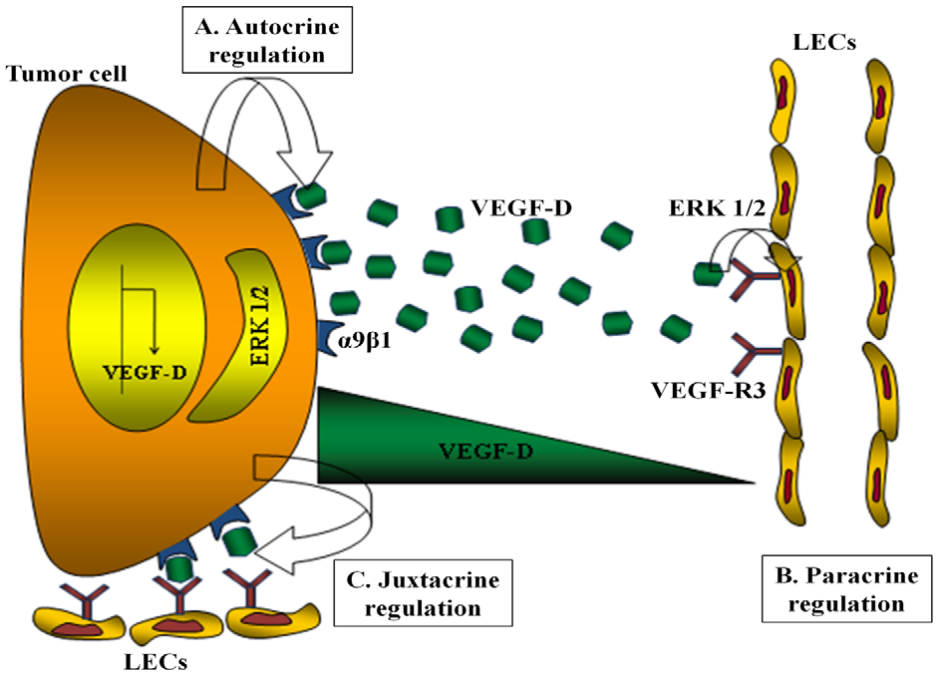
Schematic representation of the cross talk between tumor cells and lymphatic endothelial cells: (**A**) In autocrine regulation, VEGF-D produced by 468LN breast cancer cell line binds to α9β1 integrin cell surface receptor to induce the migration and invasion of cancer cell via Erk signaling. (**B**) In paracrine regulation, VEGF-D secreted by 468LN cells binds to VEGF-R3 on lymphatic endothelial cell (LEC) surface and induces migration and tube formation by the LEC via Erk signaling. (**C**) Binding of VEGF-D to α9β1 integrin located on the cancer cell membrane still leaves its binding site for VEGF-R3 located on the LEC membrane unoccupied. This leads to dual signaling by this ligand for both tumor and endothelial cells in a highly localised manner, defined as juxtacrine regulation.

We have employed multiple *in vitro* and *in vivo* approaches, which complement one another for unveiling mechanisms. *In vitro* approaches included proliferation, migration, invasion assays for tumor cells revealing autocrine stimulation by endogenous VEGF-D. To test and dissect the paracrine and juxtacrine roles of VEGF-D produced in the tumor micro-environment, we employed HMVEC-dLy cells in a number of *in vitro* assays: tube formation, migration and invasion of the ECM, which are recognized as key steps for angiogenesis and lymphangiogenesis. In all cases the stimulation provided by tumor cell-conditioned medium or co-culture with tumor cells was attenuated by knocking down VEGF-D, and the stimulation could be restored with exogenous VEGF-D. In co-culture experiments the role of tumor cell-derived VEGF-D in accelerating tubulogenesis by HMVEC-dLy cells can best be interpreted as a juxtacrine regulation (Fig 15). As noted in the present study, an alignment of two partners was also reported during tube formation by coculturing HUVEC cells with a brain cancer cell line [41]. We suggest that this alignment demonstrated *in vitro* is an indicator of close physical association of the partners allowing for a juxtacrine stimulation of lymphatic and blood capillary growth by recruitment of endothelial cell precursors.

The *in vivo* studies of lymphangiogenesis utilized three different approaches: Evans blue dye tracing, quantification of LYVE-1 marker at the mRNA and protein levels, and identification of lymphatic vessels by immunofluorescence. The *in vivo* studies on lymphatic metastasis utilized two different approaches: the presence of human mitochondrial marker MTCO2 and the GFP marker. Our *in vivo* results clearly indicate that VEGF-D is a key lymphangiogenic factor for lymphangiogenesis on its own and abrogation of expression of either VEGF-D or its receptor α9 integrin could completely eradicate lymphangiogenesis and lymphatic metastasis. A concomitant reduction in tumor growth by VEGF-D knock down can be attributed to the autocrine proliferation stimulating role of VEGF-D shown *in vitro*. The fact that α9 integrin knock down alone was equally efficient as VEGF-D knock down in abolishing lymphangiogenesis and lymphatic metastasis reinforces the juxtacrine role of α9β1 bound VEGF-D, which can additionally bind to VEGF-R3 expressed by lymphatic endothelial cells to stimulate the cellular steps in lymphangiogenesis (Fig 15).

In our earlier studies with human breast cancer [7], [8], [9], we identified the potential roles of COX-2, VEGF-C and a diverse family of VEGF-C binding receptors for lymphangiogenesis and lympho-vascular invasion. The present study shows that COX-2 independent overexpression of VEGF-D can play an equally important role in these events, and α9β1 integrin represents an important lymphangiogenic receptor as well as a receptor for autocrine motility in cancer cells. The migration and invasion-stimulating role of endogenous VEGF-D in 468LN cells was demonstrated by silencing VEGF-D gene. The obligatory role of α9β1 integrin as the dominant receptor of this ligand for mediating these functions was demonstrated by using α9β1 blocking antibody as well as silencing of the α9 gene in cancer cells. Furthermore, silencing of α9 was shown to suppress both native and VEGF-D stimulated Erk1/2 phosphorylation, a key signaling event in α9-VEGF-D interaction [11]. Autocrine actions of VEGF-C and VEGF-D including their ability to stimulate cellular migration has been documented in other cell types, for example, Kaposi's sarcoma cells [42] and lung cancer cells [43] in conjunction with receptors like VEGFR-2 and VEGFR-3.

Overexpression of α9β1 integrin in 468LN cells appears to provide a metastatic advantage by virtue of overexpression of at least two ligands, osteopontin and VEGF-D. This integrin (also known as VLA-9) is a key receptor involved in leukocyte migration [44], due to interaction with multiple ligands including osteopontin [45]. Since osteopontin, a metastasis promoter protein [46] and a marker of human breast cancer progression [14], [47], [48], is over-expressed in 468LN cells, this ligand may present an additional autocrine pathway for metastasis, at least in part by interaction with α9β1 via the RGD domain [14]. The important role of α9β1 as a lymphangiogenic receptor because of its binding to VEGF-C and VEGF-D has been well established [11]. This function explains why homozygous null mutation of the α9 gene causes congenital chylothorax in the murine fetus [49]. Tumoral expression of α9β1 showed a significant association with reduced overall patient survival and with distant-metastasis-free survival [50]. The ability of α9β1 to bind VEGF-A can also contribute to angiogenesis [51], [52], as also documented by reduced tumor-associated angiogenesis by α9 knock down in our *in vivo* studies. Interestingly, another α9β1 ligand tenascin, an ECM protein, was reported to be a stromal marker for malignancy in mammary epithelium [53]. Thus expression of α9β1 by breast cancer cells and presence of any of the above ligands in the tumor microenvironent could increase their migratory or invasive capacity to promote metastasis by both vascular and lymphatic routes. For example, host macrophages, which are known to be a rich source of both VEGF-C and VEGF-D during inflammation [28] as well as tumor growth [27], could have a paracrine effect on cancer cells in promoting metastastic phenotype. Indeed, we found that the conditioned medium from the VEGF-C and VEGF-D producing macrophage cell line RAW 264.7 strongly stimulated migration and invasiveness of 468LN cells, and this stimulation was largely abrogated in α9 silenced cells. Furthermore, genetic depletion of α9 completely abrogated tumor-associated lymphangiogenesis and lymphatic metastasis *in vivo* affirming the key role of this receptor in these events possibly utilising multiple ligands produced by the tumor as well as host cells.

We have previously shown that COX-2 mediated VEGF-C upregulation in human breast cancer cell lines [7], [9] confers a prometastatic phenotype by two mechanisms: increased ability for inducing lymphangiogenesis in situ [9] and an autocrine stimulation of motility by utilizing a diverse family of VEGF-C receptors [8]. VEGF-D can now be added to VEGF-C as a partner in promoting lymphatic metastasis in human breast cancer by utilizing the same mechanisms. Peritumoral lymph vessel density in invasive lobular breast cancer was correlated with VEGF-D expression by cancer cells and lymph node metastasis [54]. VEGF-D, which can bind to both VEGFR-2 and VEGFR-3, was shown to be capable of inducing both angiogenesis and lymphangogenesis leading to lymphatic metastasis in NOD/SCID mice transplanted with a tumor cell line forced to over-express VEGF-D, but not VEGF-A. The lymphatic spread by the VEGF-D expressing line could be blocked with a VEGF-D specific antibody [55]. We could block every event in tumor progression by either VEGF-D or α9 knock down, such as tumor growth, tumor associated lymphangiogenesis, angiogenesis, and lymphatic metastasis, indicating the independent as well as combined roles of the ligand and the receptor in cancer progression *in vivo*. The independent role of this receptor in the present cell line can be attributed to the paucity of expression of an alternate lymphangiogenic receptor such as VEGF-R3 (Fig 3A). The independent role of this ligand suggests the importance of the demonstrated autocrine, paracrine and proposed juxtacrine roles of VEGF-D (Fig 15). Furthermore, angiogenic ability of VEGF-D by binding to VEGF-R2 on endothelial cell precursors [55] may explain reduced tumor associated angiogenesis *in vivo* following VEGF-D knockdown. While VEGF-D expression was reported to be associated with poor prognosis in human breast cancer [56], the prognostic role of the combined expression of α9 integrin and VEGF-D in human breast cancer remains to be investigated.

In conclusion, our combined *in vitro* and *in vivo* results with the MDA-MB-468LN breast cancer cell line revealed that key events such as lymphangiogenesis, lympho-vascular invasion and lymphatic metastasis can be triggered by highly aggressive breast cancer cells themselves, in addition to host cells within the tumor micro-environment, involving autocrine, paracrine and juxtacrine pathways.

### Ethics Statement

*N/A*

## Materials and Methods

### Cell lines

Human breast cancer cell lines MDA-MB-468GFP and MDA-MB-468LN were used from the original stock shortly after their generation [10]. Human MDA-MB-231, SK-BR-3, Hs578T, T47D, MCF-7 breast cancer cell lines and mouse macrophage RAW 264.7 cell line were purchased from ATCC (Manassas, VA). Human MCF7-COX-2 breast cancer cell line was generated in our laboratory by stable transfection of MCF-7 cells with COX-2 cDNA cloned into the eukaryotic expression vector pIRES2-EGFP (M Majumder 2011, unpublished). MDA-MB-468GFP and 468LN cells were grown in α-MEM; MDA-MB-231, Hs578T, T47D, and RAW 264.7 were maintained in DMEM (GIBCO/Invitrogen), MCF7-COX-2 and SK-BR-3 were grown in EMEM and McCoy's 5A (ATCC), respectively. All cell lines were grown as a monolayer in basal medium supplemented with 10% FBS, 50 U/ml penicillin and 50 µg/ml streptomycin (GIBCO/Invitrogen). Medium for MCF7-COX-2 cells was supplemented with 0.01 mg/ml bovine insulin and 500 µg/ml Gentamicin (Invitrogen). Primary human dermal lymphatic microvascular endothelial cells (HMVEC-dLy) (Clonetics®, LONZA), were grown in EGM®-2-MV Bulletkit® (CC-3202, LONZA), for initial expansion. Subsequent passages were done according to the manufacturer's protocol. HMVEC-dLy cells at seventh passage were used for *in vitro* tube formation, migration, and invasion assays under growth conditions as specified later.

### Transfection of cells with siRNAs and shRNA plasmids

All *in vitro* experiments were performed with transiently siRNA transfected cells and all *in vivo* experiments with shRNA plasmids stable transfected 468LN cells. Cells were grown in 6-well plates in antibiotic-free medium, plated overnight at 37^°^C, 5% CO_2_ and then transfected with 1–5 µM of either non-targeting siRNA (scrambled siRNA) or siRNA targeting VEGF-D (s5203) and α9 (s7554) (Ambion, Applied Biosystems) with Amaxa® Cell Line Nucleofector® Kit V, according to the manufacturer protocol (LONZA). The transfection efficiency was validated by qRT-PCR, western blot, and also by ELISA for VEGF-D. We established stable VEGF-D and α9 integrin knock down 468LN cell lines using shRNA plasmids (h) [VEGF-D (sc-39844-SH, α9 integrin (sc-35674-SH) and control (sc-108060), from Santa Cruz Biotechnology, CA]. After transfection (with 10 µg of respective plasmids), cells were grown in α-MEM medium supplemented with 10% FBS and antibiotic selection started 48 h post transfection with puromycin dihydrochloride 3 mg/ml (sc-108071, Santa Cruz Biotechnology, CA). After 3–4 weeks, puromycin-resistant colonies were selected and measured for VEGF-D and α9 integrin expression by qRT-PCR and western blot. The VEGF-D and α9 integrin silenced clones were named as ΔVEGF-D/468LN and Δα9/468LN and maintained in α-MEM supplemented with 10% FBS in the presence of 500 µg/ml puromycin.

### Real time RT-PCR

Total RNA was isolated from cancer cells, human lymphatic cells, tumor tissues and lymph nodes using Qiagen RNeasy (QIAGEN, Toronto). First-strand cDNA was synthesised from 2μg of total RNA using High Capacity cDNA Reverse Transcription Kit (Applied Biosystems). Semi quantitative RT-PCR was performed using primers for VEGF-A, -C, and -D, COX-2, VEGF-R2, -R3 and GAPDH [7], [9]. Quantitative (q) RT-PCR was performed for human (VEGF-D, α9, MTCO2, GAPDH) and mouse (LYVE-1 and β-actin) genes with TaqMan Gene Expression Assays (Applied Biosystems).

### Protein detection and measurement

To measure protein levels with western blot, cells, tumor and lymph node specimens were lysed with RIPA buffer containing Halt™ Protease Inhibitor Cocktail (PIERCE, Rockford IL), and 15–20 µg protein was run on 10–12% SDS-PAGE gel. Human VEGF-A, -C, -D, -R2, -R3, COX-2, α9, MTCO2 and GAPDH proteins were detected by using monoclonal anti human VEGF-A (sc-507), VEGF-C (sc-1881), VEGF-D (sc-13085), COX-2 (sc-1747) from Santa Cruz Biotechnology, CA,VEGF-R2 (55B11) and VEGF-R3 (C28G5) from Cell Signaling Technology (Danvers, MA), integrin α9 (MAB4574) (R&D Systems, MN), GAPDH (MAB374) from Millipore (Billerica, MA) antibodies and MTCO2 (ab3298) from Abcam, US. Mouse β-actin and LYVE-1 proteins were detected by using anti mouse β-actin (sc-47778) from Santa Cruz and LYVE-1 (cat. no.11–034) from Angiobio, US. Erk1/2 and phospho Erk1/2 proteins were measured using monoclonal anti human antibodies (9102 and 9101S) from Cell Signaling. IRDye 800 conjugated anti-rabbit IgG (VEGF-A, -D, -R2, -R3, Erk1/2, pErk1/2, LYVE-1), anti-goat IgG (VEGF-C, COX-2) (Rockland Immunochemicals, Gilbertsville, PA) and Alexa 680 conjugated anti-mouse IgG (Molecular Probes/Invitrogen) were used as secondary antibodies. Immunoblots were quantified with Odyssey Infrared Imaging System (LI-COR Biosciences). Secreted VEGF-C and -D proteins were measured with Quantikine® Human VEGF-C (DVEC00) and -D (DVED00) Immunoassay (R&D Systems, MN).

### Migration, invasion and proliferation assay

Cellular migration and invasion (at 24 h and 48 h respectively) were measured as reported earlier [9], [57] with minor modifications. Near confluent cells were serum starved overnight. Then, 4×10^4^ cells/ml cells in an antibiotic-free medium and respective treatments were placed in the upper insert and allowed to migrate or invade; the bottom well was filled with the same solution used to re-suspend the cells (for native migration) or solutions inclusive of treatments (for experimental migration). The presence of 2% FBS to the basal medium served as positive controls for stimulation. The assembled cell culture insert chambers were then incubated at 37°C, 5% CO_2_ for 24 h for migration and 48 h for invasion. On completion of the incubation, the upper surfaces of the membranes were wiped gently with cotton swabs to remove non-migratory cells. The use of time points for scoring the number of migrating or invading cells at the bottom-side of membranes were based on pilot experiments, in which both events reached their peaks under native or serum stimulated conditions. The membranes were then fixed and stained, and the absolute number of migrating cells was scored visually using light microscope (40X objectives). Effects of exogenous recombinant VEGF-D (rVEGF-D) (2.5 ng/ml) (622-VD-005, R&D Systems) on both HMVEC-dLy and cancer cells, monoclonal anti human α9/β1 blocking antibody (sc-59969) (Santa Cruz, CA) on cancer cells, and Erk inhibitor (U0126 from Cell Signaling) on HMVEC-dLy cells were tested at the migration and invasion levels. In some experiments measuring migration of HMVEC-dLy cells, cell free supernatant (or conditioned medium) of 468GFP or 468LN cells were included in the bottom chamber as a source of stimulus. Each treatment was performed in triplicate or quadruplicate. Breast cancer cell proliferation was measured using Cell Proliferation ELISA kit (Roche Applied Science, IN). Briefly, cells were grown in 96-well tissue-culture microplates (2×10^4^ cells/well) for 24 h in complete medium and incubated with BrdU for another 4 h. After removal of BrdU containing medium, cells were fixed and anti-BrdU-POD antibody was added, which recognises the BrdU incorporated into the newly synthesized cellular DNA. The immune complexes were detected by the subsequent substrate reaction. The reaction product was quantified by measuring the absorbance using a scanning multi-well spectrophotometer (Tecan, Infinite M200).

### *In vitro* lymphangiogenesis (tube formation) assay

Semi confluent HMVEC-dLy cells were grown on 12-well tissue culture plates (2×10^5^ cells/per well) evenly coated with 0.1 ml/well growth factor-reduced Matrigel (BD Biosciences, San Jose, CA), diluted with serum free medium in 1∶1 ratio. The time course of tube formation in serum free medium (SFM) under control or experimental conditions was recorded with a fluorescence microscope (Leica DFC340FX). Vascular-like channels were quantified by counting the total number of complete tubes and the number of branching points in the same field at different (x5 and x10) objectives using ImageJ software. When cells connected to each other to complete a capillary like structure was called a tube, whereas, each point connecting rows of cells was called a branching point [58]. Five randomly selected fields of view with x10 objective were photographed in each well. The average of five fields was taken as the value for each sample. Finally data from triplicate or quadruplicate experiments were expressed as a percentage of the respective controls.

### Mice

Six weeks old athymic nude female mice (Hsd: Athymic Nude-*Foxn1^nu^/Foxn1^+^*, Harlan, IN), were allowed to acclimatize for 2–3 weeks, maintained on standard mouse chow and tap water (unless otherwise indicated) on a 12 h light/dark cycle, and treated in accordance with the guidelines set by the Canadian Council on Animal Care.

### Tumor transplantation and measurements of tumor progression

Tumor growth, tumor-associated angiogenesis, lymphangiogenesis and lymphatic metastasis were measured with an *in vivo* assay devised in our laboratory [39] in which tumor cells suspended in growth factor reduced Matrigel were implanted S.C., allowing ingrowths of capillaries and lymphatics from pre-existing vasculature. Originally devised for measuring the kinetics of tumor-associated angiogenesis [39], our pilot studies revealed that this *in vivo* assay was also exquisitely suited for measuring tumor-associated lymphangiogenesis. Mice were randomized into control and experimental groups and received subcutaneous implants of 20×10^4^ either 468LN or ΔVEGF-D/468LN or Δα9/468LN cells suspended in growth factor reduced Matrigel (3.5 mg Matrigel in 0.5 ml αMEM) in both inguinal and axillary mammary regions (each animal four points). Some animals received implants of Matrigel alone, to serve as negative controls for angiogenesis or lymphangiogenesis and lymphatic metastasis. The tumor volumes measured externally with calliper at 2 days intervals (computed as 0.5×a^2^ b, from the minimum (a) and maximum (b) diameters) as well as the weights of fresh unfixed tumors retrieved at sacrifice. Mice were sacrificed by exposure to halothane followed by cervical dislocation, at day 20 (n = 4 for each cell line). Upon retrieval of the Matrigel implants (4 per mouse) at day 20, gross morphology of tumor and tumor draining lymph nodes were photographed. Tumors and tumor-draining lymph nodes were then sliced in three pieces, one for frozen section for further histological or immuno-histological analysis, other parts for RNA and protein extraction respectively. Implants of Matrigel alone at four points in one mouse remained unchanged in size, serving as negative control.

### Evans Blue visualization of lymphatic vessels *in vivo*

After the tumors had grown to between 0.5 and 1 cm in diameter, the mice were anesthetised with isoflurane (Pharmaceutical Partners Of Canada Inc., ON) and the tumors were exposed. To identify the lymphatics in nude mice, 2 µl of 4% Evan's blue dye (Sigma-Aldrich, CA) in PBS were injected 2 cm apart from the tumor at a single point, allowing a visual tracing of lymphatics into both 468LN and ΔVEGF-D/468LN tumors. Images were acquired with a digital camera (Nikon, D-90) at 5 minutes and then consecutive pictures were taken with dissection microscope (Nikon SMZ1500) after 10–25 minutes to identify lymphatic vessels impinging on the tumor.

### Measurements of angiogenesis and lymphangiogenesis

Eight micrometer (µm) thick frozen sections of Matrigel implants were fixed in methanol for 3 minutes and then in acetone for 3 minutes, both at 4°C. All of the subsequent steps were performed at room temperature. Sections were air-dried for 1h, rehydrated for 10 minutes in PBS, followed by treatment with 2% BSA in PBS for 20 minutes to block non-specific antibody binding. Sections were then incubated for 1 h with rabbit anti-mouse LYVE-1 (Upstate biotechnologies, Lake Placid, NY; 1∶200) and rat anti-mouse CD31 (Caltag Laboratories, Advanced medical sciences, Bangkok, Thailand, 1∶1000) antibodies, followed by normal mouse serum (1%) for 10 minutes to block non-specific cross-reactivity of secondary antibodies with mouse proteins. Nuclei were stained with DAPI (4, 6-diamidine-2-phenylindole hydrochloride, 1∶10,000) from Invitrogen. Sections were then incubated for 30 minutes with goat anti-rabbit Alexa Fluor 488 and donkey anti-rat Alexa Fluor 594 secondary antibodies (Molecular Probes, Burlington, ON, Canada; 1∶1000), washed with PBS, drained, and mounted with Vectashield solution (Vector Laboratories, Burlington, ON). The sections from all tumors were first scanned at low magnification (250×) under a BX51 microscope (Olympus) to identify the most vascular areas of the tumor. Microvessel density (MVD) representing blood vessels and lymphatic vessel density (LVD) were assessed in serial immunostained sections of frozen tissues as reported [39], [40], with the following modification. Three hotspots per tumor (4 tumor per mouse×4mice×3 spots or 48/group) were examined at 40×magnification [40], and scored after setting the threshold for background, using the Image-J software (NIH, USA). The mean values for MVD and LVD were computed as the staining indices for CD31 (red) and LYVE-1 (red), respectively.

### Indirect quantification of lymphangiogenesis

Total RNA and protein were extracted from tumors. Presence of murine lymphatic endothelial cells in tumors resulting from transplantation of human cancer cells were quantified with Taqman Gene expression assay using mouse specific probe for the lymphatic endothelial cell marker LYVE-1 (Mm00475056_m1, cat no. 4331182, Applied Biosystems), and western blot analysis with monoclonal antibody to mouse LYVE-1 (cat no. 11–034, Angiobio, US).

### Quantification of lymphatic metastasis

Total RNA and protein were extracted from tumor draining lymph nodes. Presence of human cancer cells in mouse lymph nodes was quantified with Taqman Gene expression assay with human mitochondrial gene (*MT-CO2*) specific probe (Hs02596865_g1, Gene ID 4513, Applied Biosystems), and monoclonal antibody to MTCO2 (1–2 µg/ml; ab3298; Abcam, US) that reacts specifically with a 60 kDa non-glycosylated protein component of human mitochondria. In addition using frozen sections of draining lymph nodes, green 468LN cells (resulting from the GFP marker) were identified with fluorescence microscopy.

### Statistics

All statistical analyses were performed using GraphPad Software (QuickCalcs). Data were analysed by two way ANOVA and Student's T-test considering P<0.05 as an indicator of significant difference between means.

## Supporting Information

Figure S1 Compared to 468GFP cells, 468LN cells were significantly more proliferative. (B) Representative images of migratory 468GFP and 468LN cells in SFM. (C) 468LN cells expressed a significantly higher level of VEGF-D mRNA measured with qRT-PCR. (D) Representative images of migratory 468GFP and 468LN cells in the presence of rVEGF-D. Images were obtained under 40X objective after 24 h incubation.
(TIF)

Figure S2 Representative images of migratory 468LN cells in SFM and before and after VEGF-D knock down. Images were obtained under 40X objective after 24 h incubation.
(TIF)

Figure S3 Migration pictures of pictures of 468LN cells showing results after α9 integrin knock down in SFM, in the presence of rVEGF-D or FBS. Images were captured with 40X objective after 24 h incubation.
(TIF)

Figure S4 Representative migration of pictures of 468LN cells in SFM and in the presence of RAW cell conditioned medium (CM) before and after α9 integrin knock down. Images were captured with 40X objective.
(TIF)

Figure S5 Migration pictures of HMVEC-dLy cells in presence of 468GFP, 468LN cells, VEGF-D knocked down 468LN cells and after addition of exogenous rVEGF-D. Images were captured with 20X objective.
(TIF)

Figure S6 Migration pictures of rVEGF-D stimulated HMVEC-dLy cells and inhibition of migration of the same cells in response to Erk inhibitor U0126 at different concentrations. Inhibition could not be retrieved with exogenous rVEGF-D. Images were captured with 40X objective.
(TIF)

Figure S7 **Stable knock down of α9 integrin and VEGF-D with shRNA plasmid:** Stable knock down of both α9 integrin and VEGF-D in 468LN cells was confirmed at protein level. Western blots showing 60–70% knock down of α9 integrin and VEGF-D compared to shRNA control knock down. The stable cell lines were named as Δα9/468LN and ΔVEGF-D/468LN for α9 integrin and VEGF-D knock down respectively.
(TIF)

Figure S8 **Estimation of 468LN cells metastasizing to the tumor draining lymph nodes by real time PCR:** 468LN cells were plated overnight for complete attachment. And then one million cells harvested to extract RNA. Then cDNA was synthesised with 2 µg of total RNA. Next, serial dilutions (1∶5) of cDNAs were prepared using the nuclease free water. Then real-time RT-PCR was performed with all diluted 468LN cDNAs. This was done concurrently with cDNAs derived with RNA extracted from lymph nodes. The Ct value obtained with the lymph node was then converted to the number of tumor cells from the standard plot.
(TIF)

Figure S9 Screening of different breast cancer cell lines for VEGF-D (**A**) and for α9 integrin (**B**) at mRNA levels with qRT-PCR and (**C**) protein levels with western blot. Expression of mRNA levels in different cell lines were quantitated relative to the expression in MCF-7 cells and presented in a log scale. Data are expressed as mean ± SE for replicate values, ** p<0.001.
(TIF)
