# Supplementary figures and images for: Co-Expression of α9β1 Integrin and VEGF-D Confers Lymphatic Metastatic Ability to a Human Breast Cancer Cell Line MDA-MB-468LN
Source: PLoS One. 2012 Apr 24;7(4):e35094. doi: 10.1371/journal.pone.0035094 (PMC3335831; doi:10.1371/journal.pone.0035094)

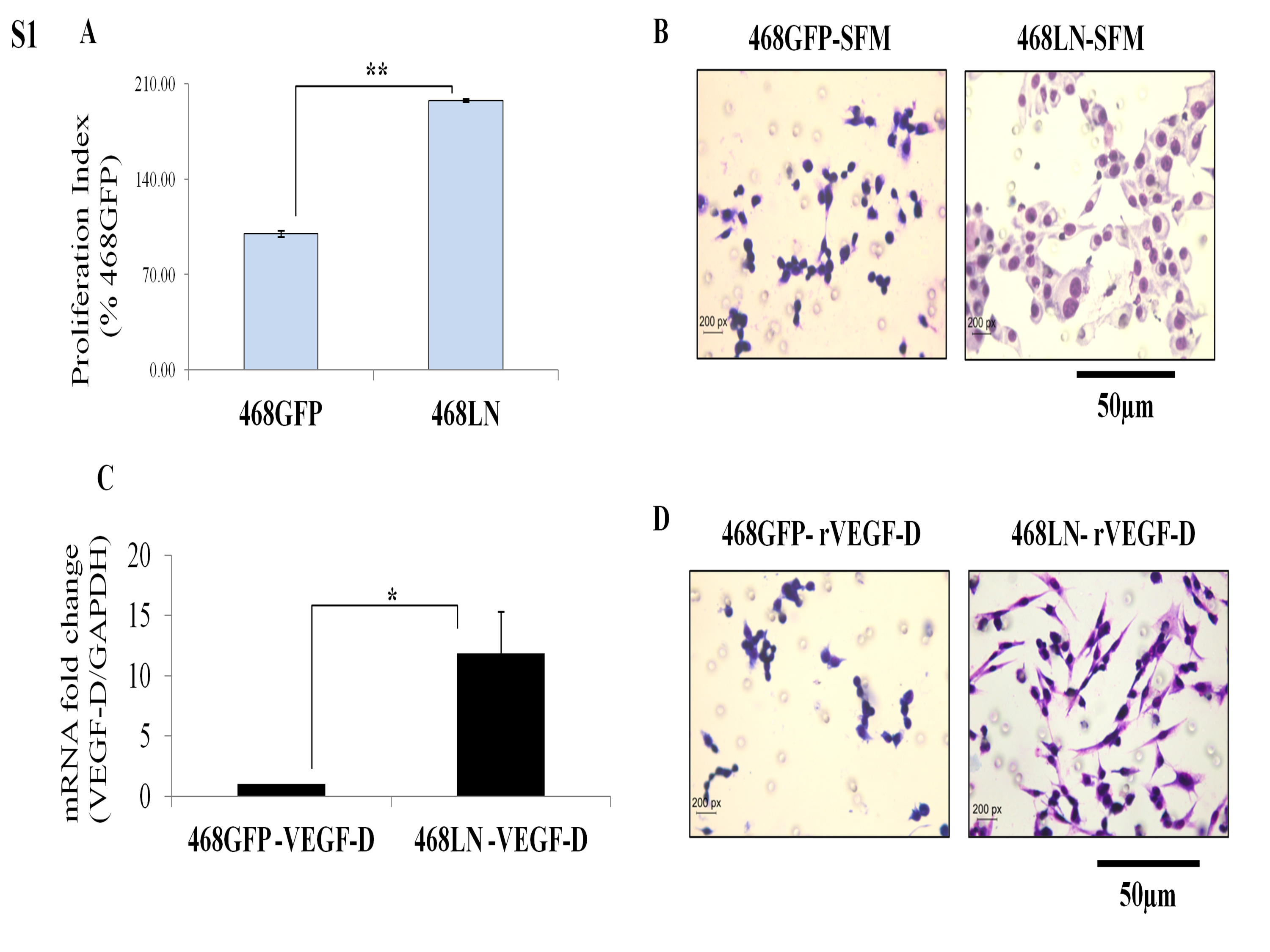

Supplement: Figure S1 — Compared to 468GFP cells, 468LN cells were significantly more proliferative. (B) Representative images of migratory 468GFP and 468LN cells in SFM. (C) 468LN cells expressed a significantly higher level of VEGF-D mRNA measured with qRT-PCR. (D) Representative images of migratory 468GFP and 468LN cells in the presence of rVEGF-D. Images were obtained under 40X objective after 24 h incubation. (TIF) [file pone.0035094.s001.tif]

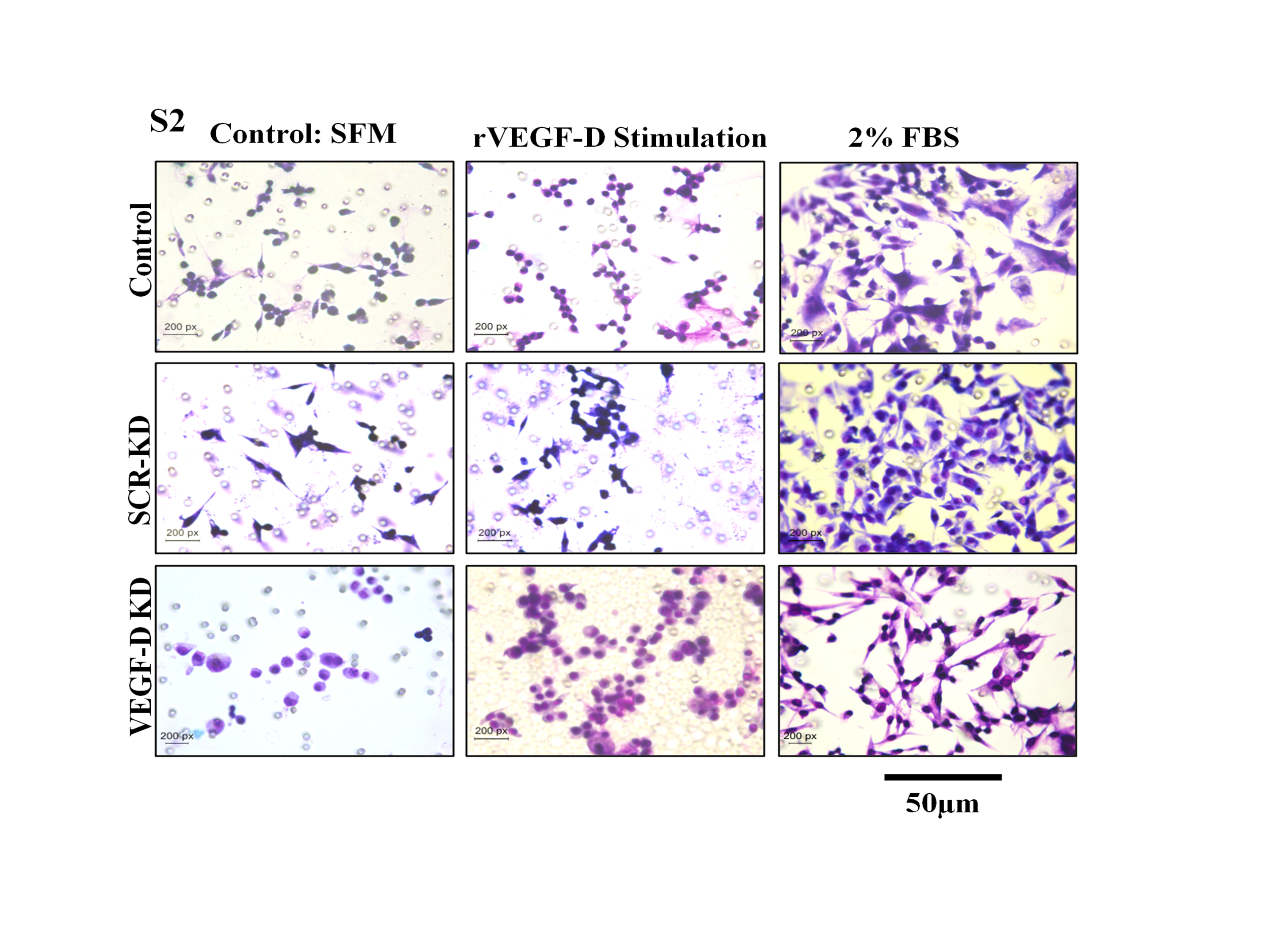

Supplement: Figure S2 — Representative images of migratory 468LN cells in SFM and before and after VEGF-D knock down. Images were obtained under 40X objective after 24 h incubation. (TIF) [file pone.0035094.s002.tif]

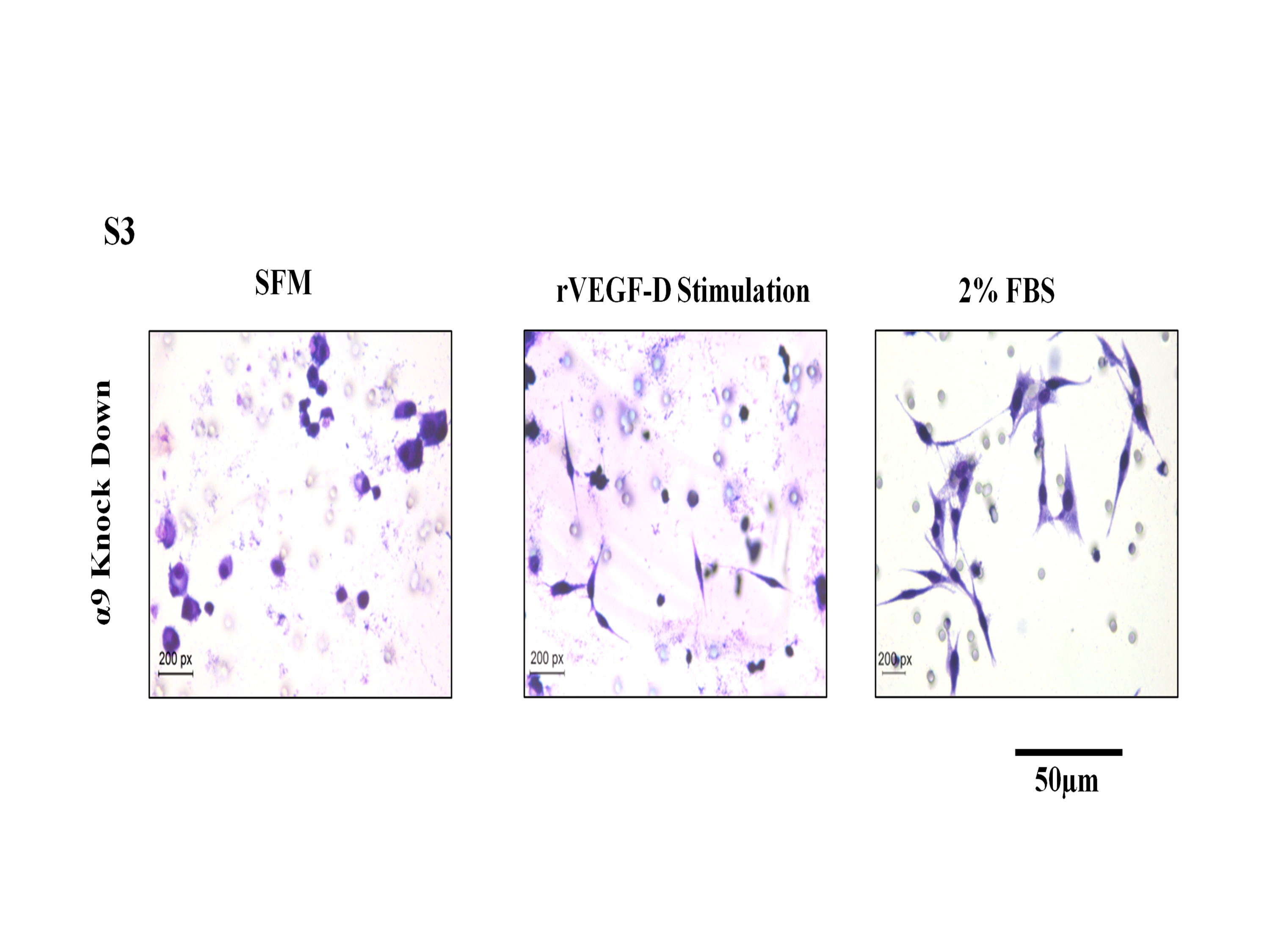

Supplement: Figure S3 — Migration pictures of pictures of 468LN cells showing results after α9 integrin knock down in SFM, in the presence of rVEGF-D or FBS. Images were captured with 40X objective after 24 h incubation. (TIF) [file pone.0035094.s003.tif]

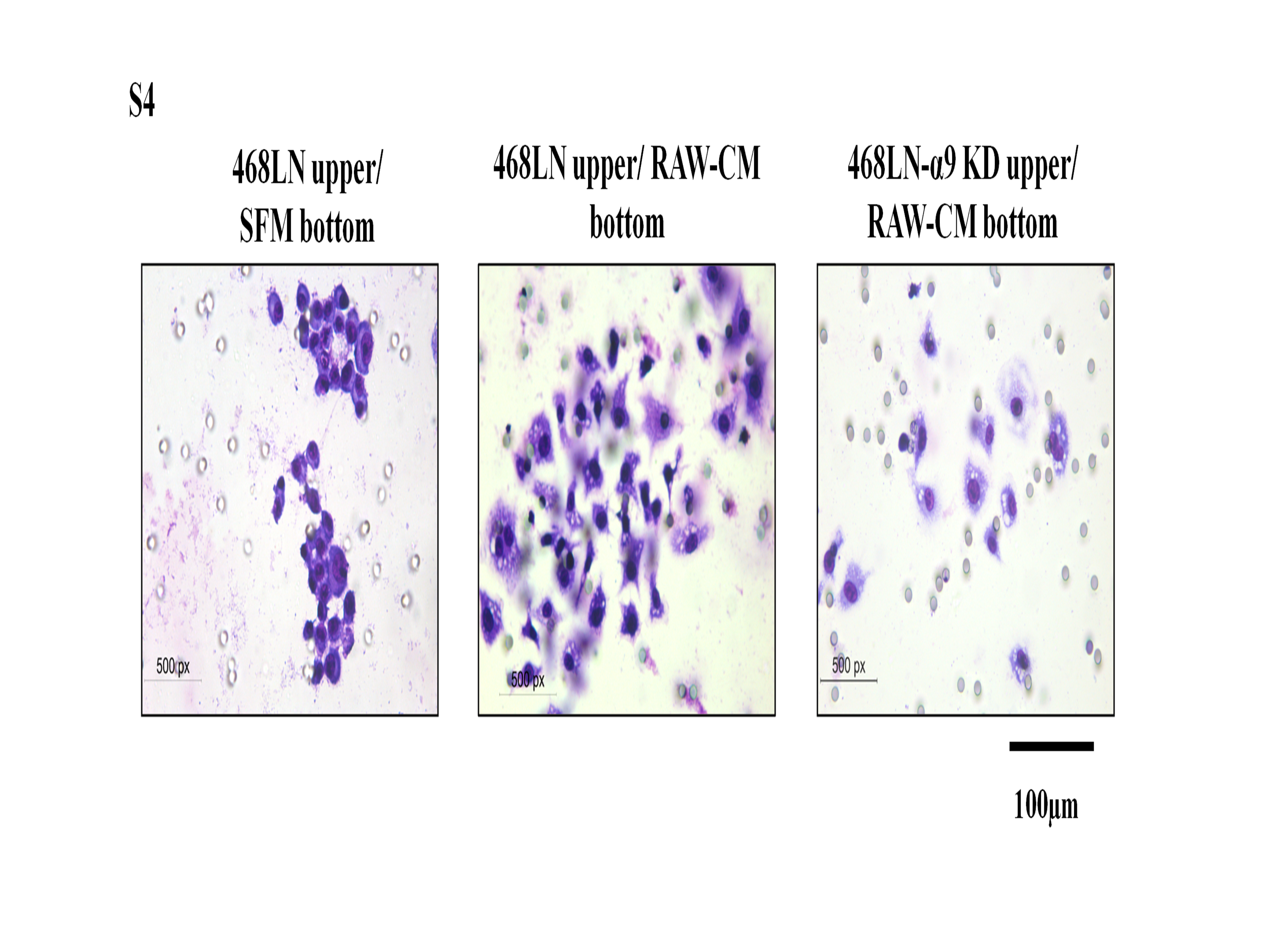

Supplement: Figure S4 — Representative migration of pictures of 468LN cells in SFM and in the presence of RAW cell conditioned medium (CM) before and after α9 integrin knock down. Images were captured with 40X objective. (TIF) [file pone.0035094.s004.tif]

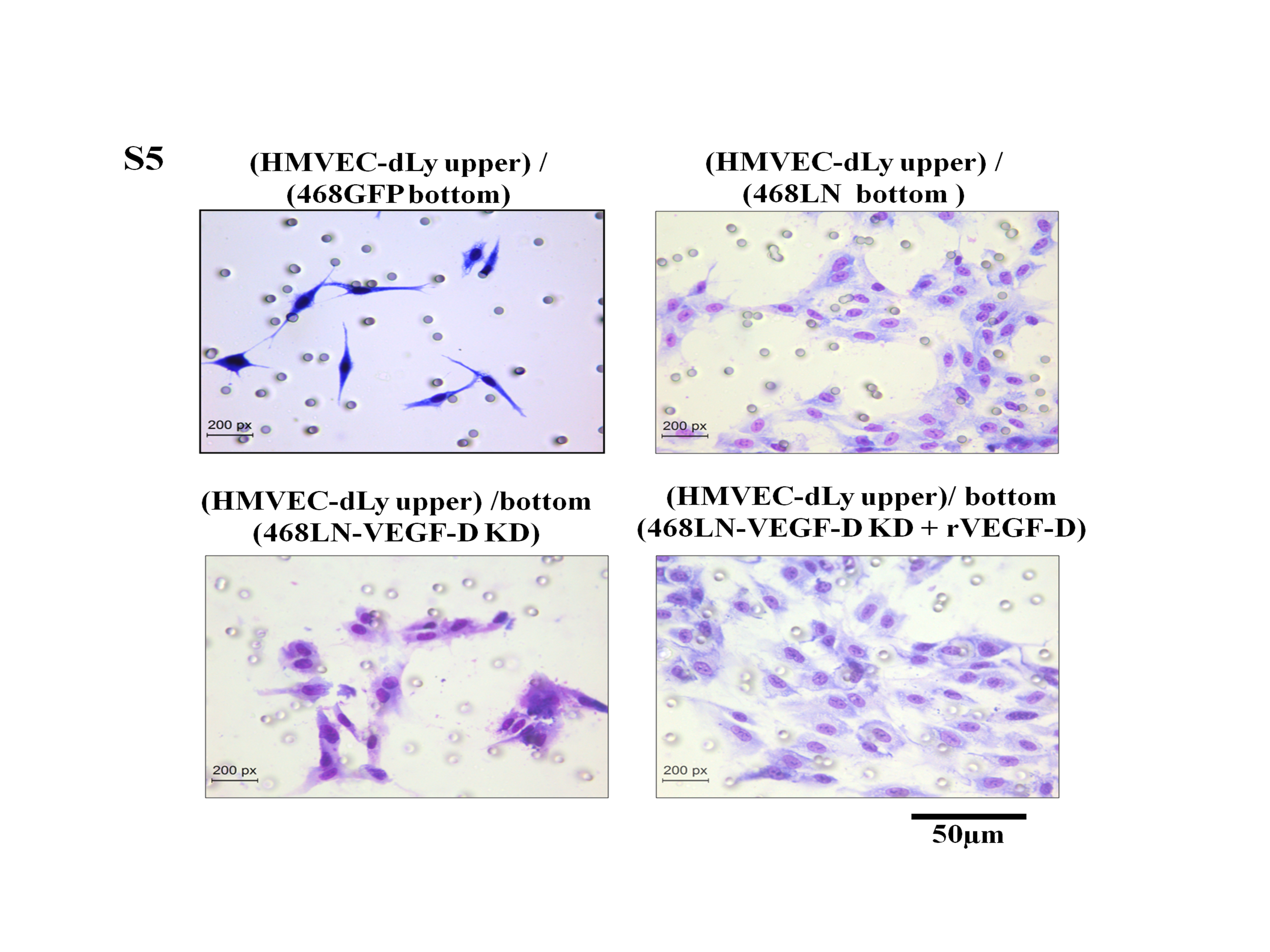

Supplement: Figure S5 — Migration pictures of HMVEC-dLy cells in presence of 468GFP, 468LN cells, VEGF-D knocked down 468LN cells and after addition of exogenous rVEGF-D. Images were captured with 20X objective. (TIF) [file pone.0035094.s005.tif]

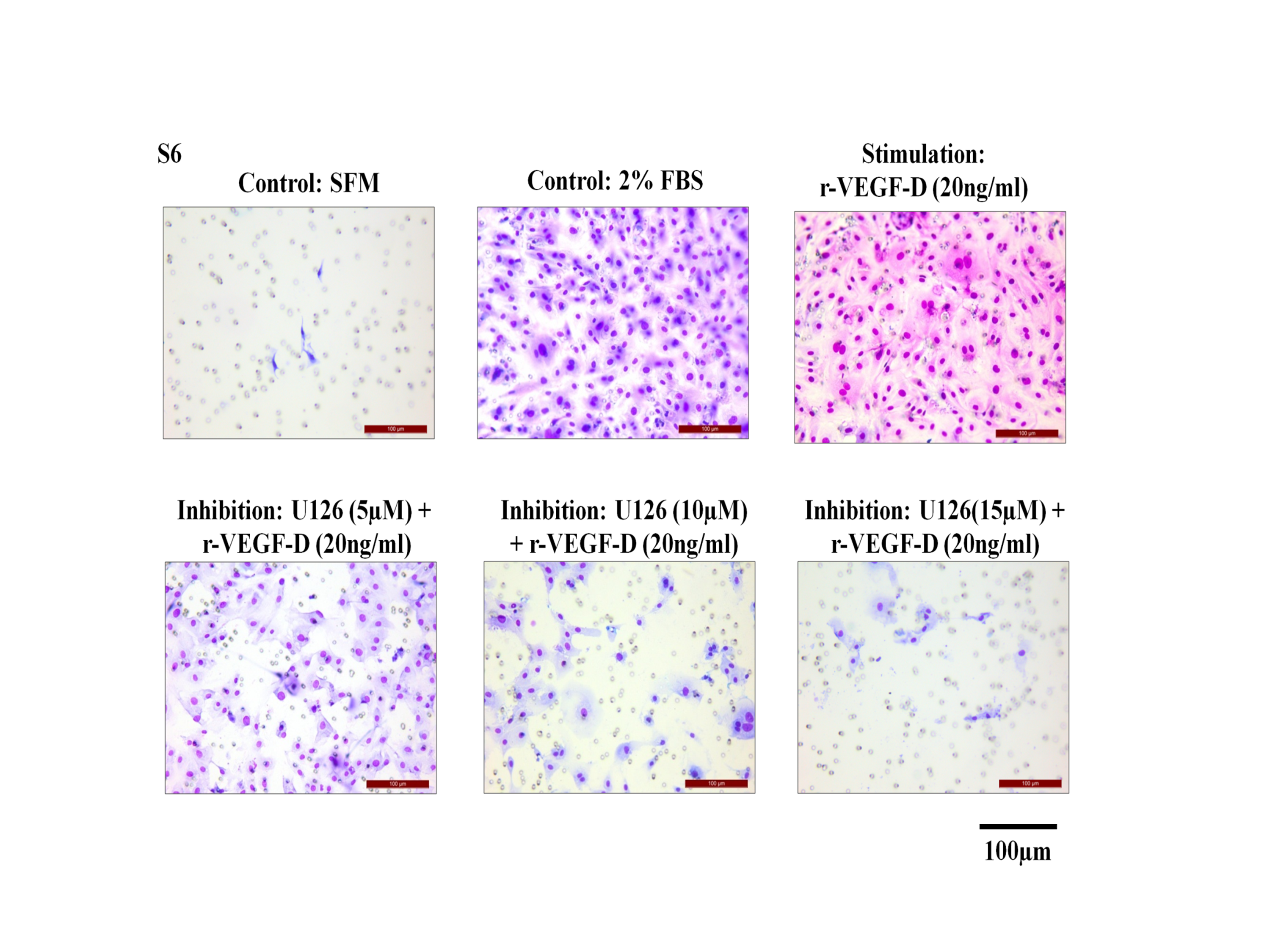

Supplement: Figure S6 — Migration pictures of rVEGF-D stimulated HMVEC-dLy cells and inhibition of migration of the same cells in response to Erk inhibitor U0126 at different concentrations. Inhibition could not be retrieved with exogenous rVEGF-D. Images were captured with 40X objective. (TIF) [file pone.0035094.s006.tif]

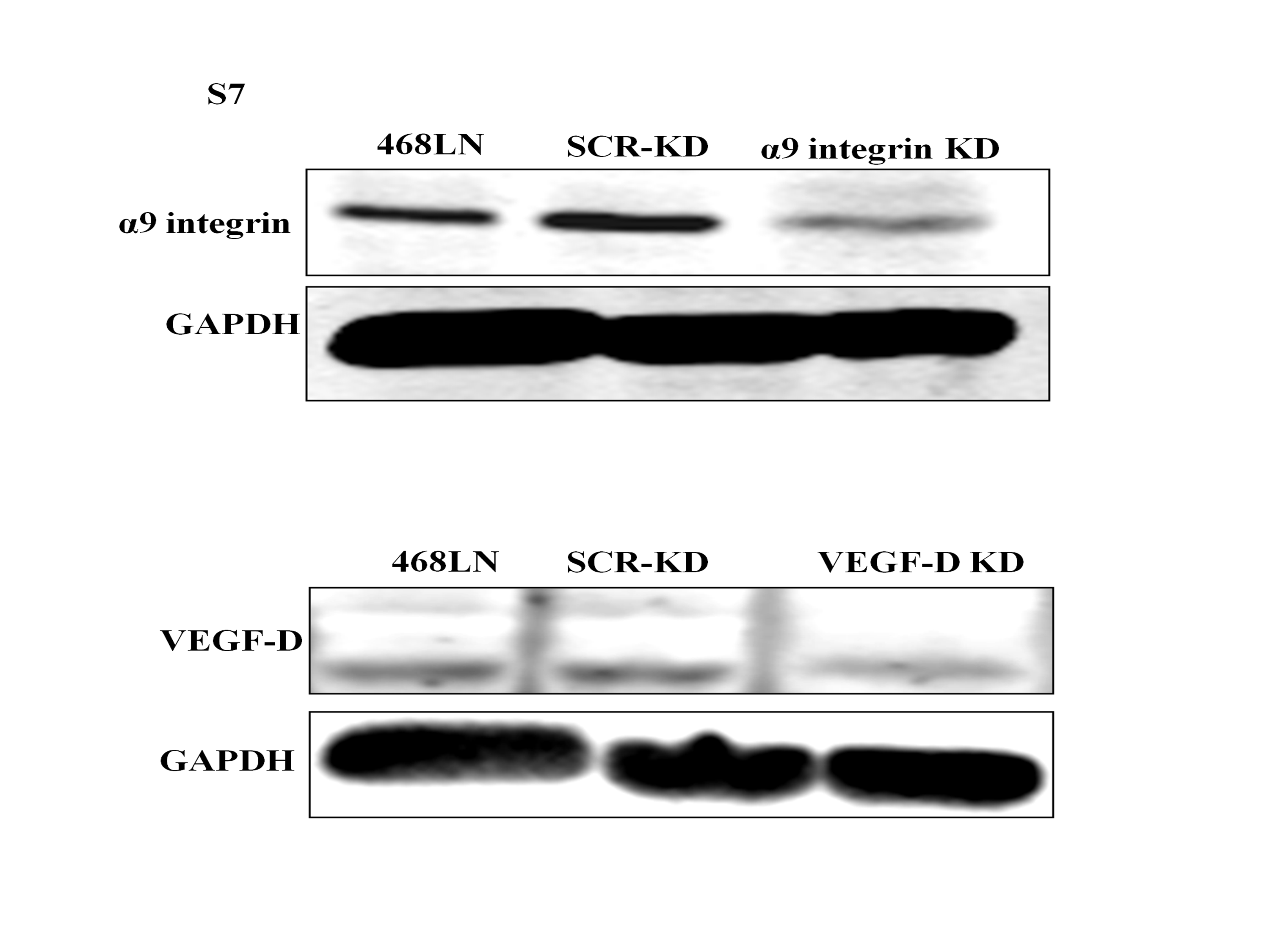

Supplement: Figure S7 — Stable knock down of α9 integrin and VEGF-D with shRNA plasmid: Stable knock down of both α9 integrin and VEGF-D in 468LN cells was confirmed at protein level. Western blots showing 60–70% knock down of α9 integrin and VEGF-D compared to shRNA control knock down. The stable cell lines were named as Δα9/468LN and ΔVEGF-D/468LN for α9 integrin and VEGF-D knock down respectively. (TIF) [file pone.0035094.s007.tif]

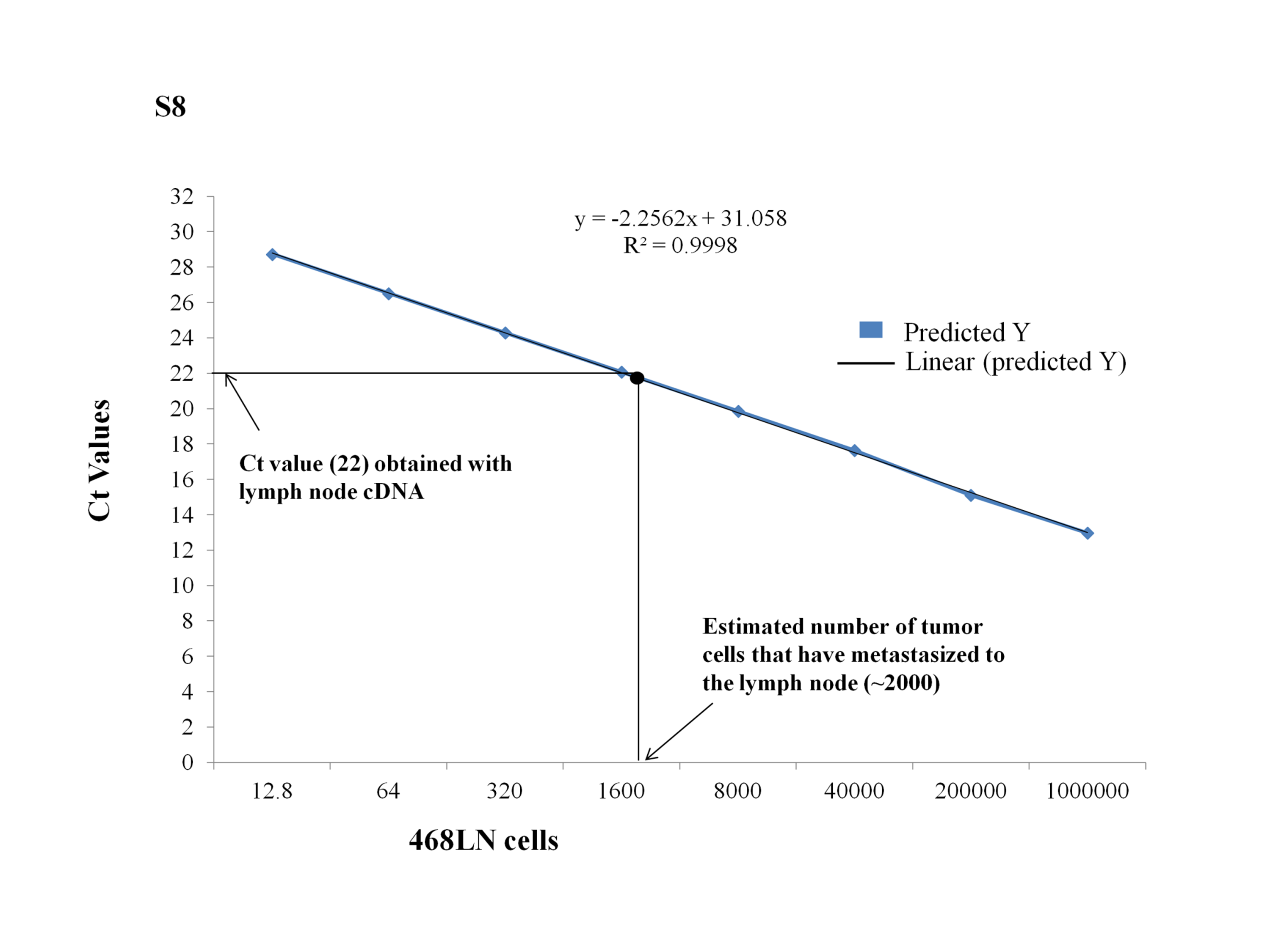

Supplement: Figure S8 — Estimation of 468LN cells metastasizing to the tumor draining lymph nodes by real time PCR: 468LN cells were plated overnight for complete attachment. And then one million cells harvested to extract RNA. Then cDNA was synthesised with 2 µg of total RNA. Next, serial dilutions (1∶5) of cDNAs were prepared using the nuclease free water. Then real-time RT-PCR was performed with all diluted 468LN cDNAs. This was done concurrently with cDNAs derived with RNA extracted from lymph nodes. The Ct value obtained with the lymph node was then converted to the number of tumor cells from the standard plot. (TIF) [file pone.0035094.s008.tif]

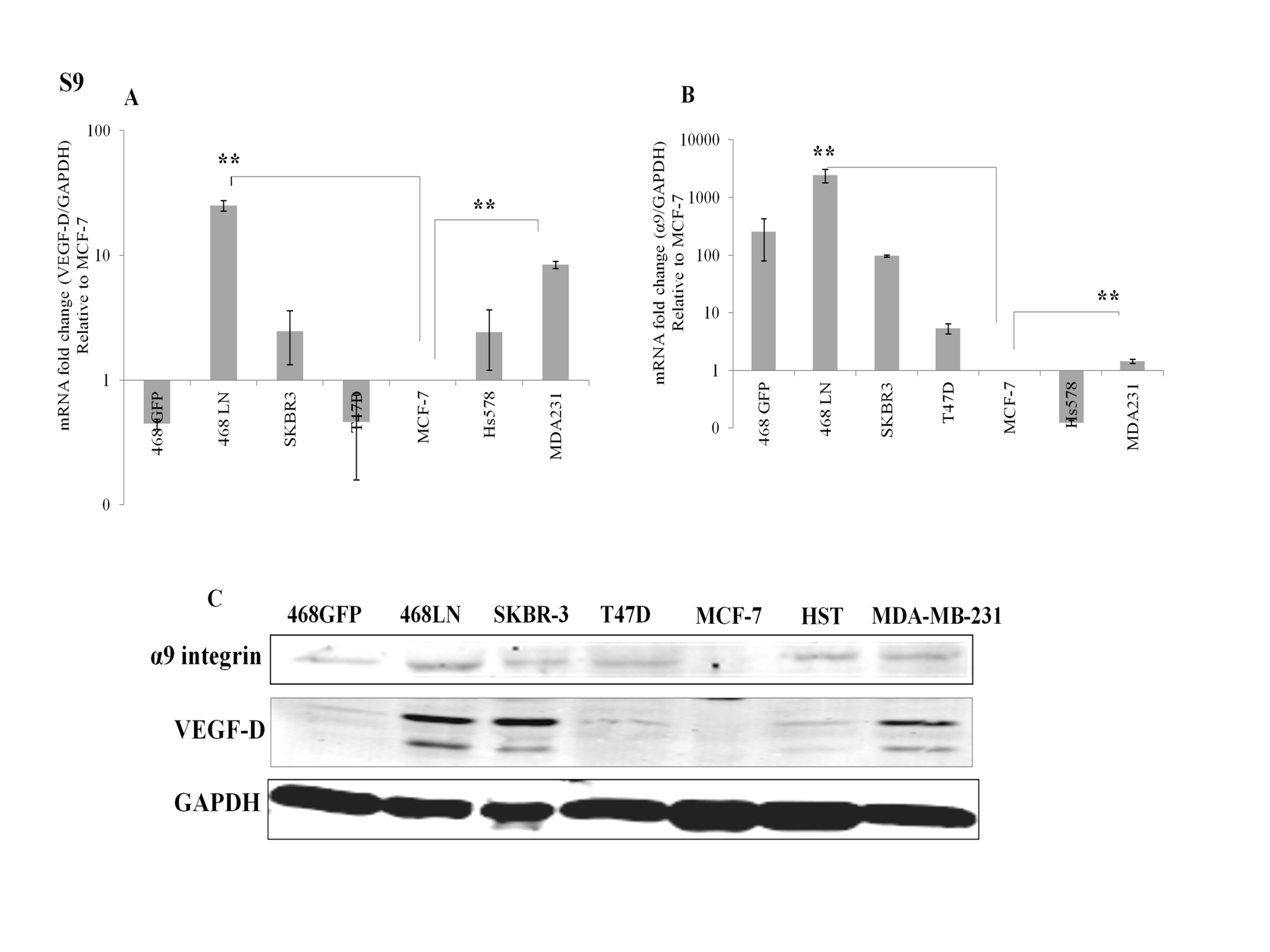

Supplement: Figure S9 — Screening of different breast cancer cell lines for VEGF-D (A) and for α9 integrin (B) at mRNA levels with qRT-PCR and (C) protein levels with western blot. Expression of mRNA levels in different cell lines were quantitated relative to the expression in MCF-7 cells and presented in a log scale. Data are expressed as mean ± SE for replicate values, ** p<0.001. (TIF) [file pone.0035094.s009.tif]
